# Heterologous prime-boost vaccination drives early maturation of HIV broadly neutralizing antibody precursors in humanized mice

**DOI:** 10.1126/scitranslmed.adn0223

**Published:** 2024-05-22

**Authors:** Christopher A. Cottrell, Xiaozhen Hu, Jeong Hyun Lee, Patrick Skog, Sai Luo, Claudia T. Flynn, Katherine R. McKenney, Jonathan Hurtado, Oleksandr Kalyuzhniy, Alessia Liguori, Jordan R. Willis, Elise Landais, Sebastian Raemisch, Xuejun Chen, Sabyasachi Baboo, Sunny Himansu, Jolene K. Diedrich, Hongying Duan, Cheng Cheng, Torben Schiffner, Daniel L.V. Bader, Daniel W. Kulp, Ryan Tingle, Erik Georgeson, Saman Eskandarzadeh, Nushin Alavi, Danny Lu, Troy Sincomb, Michael Kubitz, Tina-Marie Mullen, John R. Yates, James C. Paulson, John R. Mascola, Frederick W. Alt, Bryan Briney, Devin Sok, William R. Schief

**Affiliations:** 1 Department of Immunology and Microbial Science, The Scripps Research Institute, La Jolla, CA 92037, USA; 2 Center for HIV/AIDS Vaccine Development, The Scripps Research Institute, La Jolla, CA 92037, USA; 3 IAVI Neutralizing Antibody Center, The Scripps Research Institute, La Jolla, CA 92037, USA; 4 Moderna Therapeutics, Cambridge, MA 02139, USA; 5 HHMI, Boston Children’s Hospital, Boston, MA 02115, USA; 6 Program in Cellular and Molecular Medicine, Boston Children’s Hospital, Boston, MA 02115, USA; 7 Department of Genetics, Harvard Medical School, Boston, MA 02115, USA; 8 Vaccine Research Center, National Institute of Allergy and Infectious Diseases, National Institutes of Health, Bethesda, MD 20892, USA; 9 Department of Molecular Medicine, The Scripps Research Institute, La Jolla, CA 92037, USA; 10 The Ragon Institute of Massachusetts General Hospital, Massachusetts Institute of Technology and Harvard University, Cambridge, MA 02139, USA

## Abstract

A protective human immunodeficiency virus (HIV) vaccine will likely need to induce broadly neutralizing antibodies (bnAbs). Vaccination with the germline-targeting immunogen eOD-GT8 60mer adjuvanted with AS01_B_ was found to induce VRC01-class bnAb precursors in 97% of vaccine recipients in the IAVI G001 phase 1 clinical trial; however, heterologous boost immunizations with antigens more similar to the native glycoprotein will be required to induce bnAbs. Therefore, we designed core-g28v2 60mer, a nanoparticle immunogen to be used as a first boost following eOD-GT8 60mer priming. We found, using a humanized mouse model approximating human conditions of VRC01-class precursor B cell diversity, affinity, and frequency, that both protein- and mRNA-based heterologous prime-boost regimens induced VRC01-class antibodies that gained key mutations and bound to near-native HIV envelope trimers lacking the N276 glycan. We further showed that VRC01-class antibodies induced by mRNA-based regimens could neutralize pseudoviruses lacking the N276 glycan. These results demonstrated that heterologous boosting can drive maturation toward VRC01-class bnAb development and supported the initiation of the IAVI G002 phase 1 trial testing mRNA-encoded nanoparticle prime-boost regimens.

## One Sentence Summary

An HIV vaccine first-boost candidate immunogen promoted maturation of VRC01-class antibodies in a humanized mouse model, supporting clinical testing.

## Editor’s Summary

Boosting HIV Antibodies. For a vaccine regimen to be effective against human immunodeficiency virus (HIV) infection, it will need to elicit broadly neutralizing antibodies (bnAbs). However, strategies to elicit bnAbs in humans with a vaccine have not been successful to date. A different strategy, germline-targeting vaccine design, relies on a priming immunogen to first induce bnAb precursors and then a series of heterologous boosters to drive or shepherd the maturation of bnAb precursors to produce bnAbs. The first step in this process, elicitation of bnAb precursors, has proven successful in clinical trials. Here, Cottrell *et al.* take the next step by designing and testing the first booster immunogen in the shepherding process. Vaccination of eOD-GT8 60mer-primed humanized mice with their immunogen, core-g28v2 60mer, in either protein or mRNA form resulted in antibodies that were closer to bnAbs than those that received a placebo booster. These data support the ongoing clinical trial testing core-g28v2 60mer mRNA as a booster vaccine. –Courtney Malo

## INTRODUCTION

An effective prophylactic vaccine against human immunodeficiency virus (HIV) is needed to help prevent the 1.3 million new infections occurring each year (1, 2). HIV broadly neutralizing antibodies (bnAbs), which bind partially conserved surface patches (epitopes) on the highly variable HIV envelope glycoprotein (Env), can provide sterilizing protection in non-human primate models (3) and can protect humans against infection (4, 5). Hence, bnAb elicitation is widely considered essential for an effective HIV vaccine. However, bnAb elicitation faces at least two major challenges. First, bnAb germline precursors typically have no detectable affinity for wild-type Env (6–15), which implies that wild-type Env proteins are unlikely to serve as effective priming immunogens to initiate bnAb induction. Second, most bnAbs are highly mutated from germline, with multiple mutations enabling high affinity binding to the cognate epitope, which indicates that repeated vaccination with a single antigen is unlikely to induce sufficient maturation to produce bnAbs.

Germline-targeting vaccine design (14–24) offers a potential solution to these challenges. In this vaccine strategy, a priming immunogen is designed to induce responses from diverse bnAb precursors for any single bnAb class, in order to prime the desired responses in all or most vaccine recipients. A series of booster immunogens are then designed to be successively closer in structure to the native glycoprotein, such that each boost should engage B cells produced by the prior immunization and select for additional mutation toward bnAb development. The IAVI G001 phase 1 trial provided clinical proof-of-principle for the priming step in the germline-targeting strategy: immunization with the germline-targeting priming immunogen eOD-GT8 60mer as protein with AS01_B_ adjuvant was found to induce bnAb precursors of the VRC01 class in 97% of vaccine recipients (25–27).

VRC01-class bnAbs bind the Env CD4-binding site (CD4bs) and prevent HIV Env from binding its primary receptor on human CD4^+^ T cells (12, 28–33). Prior work has shown that passively administered VRC01 IgG can protect humans from infection with diverse VRC01-sensitive viruses (4, 5), providing evidence that similar VRC01-class bnAbs elicited by vaccination can protect humans from HIV infection. The eOD-GT8 60mer is a self-assembling nanoparticle designed to bind to diverse VRC01-class naïve human precursors with substantial affinity and avidity (34) and in pre-clinical studies was shown to prime VRC01-class B cell responses in multiple knock-in and adoptive transfer mouse models (21, 22, 34–42).

The next critical test of the germline-targeting strategy is to determine whether a suitably designed first-boost immunogen can advance maturation toward bnAb development. To support use of mRNA delivery to accelerate clinical testing, pre-clinical studies with mRNA immunogens must be performed. Here, we used VRC01-class and CD4bs-specific non-VRC01-class monoclonal antibodies (mAbs) from the IAVI G001 study to aid in the design of first-boost candidates to follow eOD-GT8 60mer priming, and we evaluated different prime-boost regimens based on mRNA or adjuvanted protein in a mouse model with VRC01-class naïve precursor frequencies and affinities approximately similar to those in humans. The results supported the initiation of the IAVI G002 human clinical trial (NCT05001373) testing eOD-GT8 60mer priming and core-g28v2 60mer boosting delivered by mRNA lipid nanoparticles (LNPs).

## RESULTS

### V_H_1–2/V_K_1–33 rearranging mice harbor VRC01-class precursors with frequencies and affinities approximating those in humans.

VRC01-class antibodies are defined by their use of the human immunoglobulin heavy chain (HC) V gene alleles V_H_1–2*02 or *04 and any light chain (LC) complementarity determining region (LCDR) 3 with a length of five amino acids (aa) (14, 43). Most of the interactions between VRC01-class bnAbs and HIV Env come from the HC complementarity determining region (HCDR) 2, with the HCDR3 providing a minor supporting role (10, 30, 31), allowing VRC01-class bnAbs to have diverse HCDR3 lengths and sequences.

The V_H_1–2^JH2^/V_K_1–33^h*TdT*^ mouse (referred to as SE09 from this point forward) has the human IGHV1–2*02 and human IGHJ2*01 alleles knocked into the mouse *IGH* locus and the human IGKV1–33*01 allele knocked into the mouse *IGK* locus (fig. S1A) (41). Additionally, the SE09 mouse has a knockout of the intergenic control region 1 (IGCR1), resulting in dominant usage of the human IGHV1–2*02 allele, and a human terminal deoxynucleotidyl transferase (*TdT*) knock-in that increases the percentage of LCs with 5 aa LCDR3s (fig. S1A) (41). The knock-in genes participate in normal V(D)J recombination, resulting in a diverse repertoire of CDR3 lengths and sequences, and the human V_H_1–2 HCs and V_K_1–33 LCs are expressed in approximately 45% and 2% of naïve B cells, respectively (41). We evaluated the VRC01-class precursor frequency in the SE09 mouse model by sorting CD19^+^/IgM^+^IgD^+^/eOD-GT8^++^ splenocytes from 10 unimmunized mice and subjecting those cells to paired single-cell B cell receptor (BCR) sequencing using 10x Genomics. The frequency of VRC01-class naïve B cells was higher in the SE09 mouse (1 in 13,600) compared to humans (1 in 228,000) (fig. S1B) (16, 44, 45). The frequency of VRC01-class naïve B cells with human V_K_1–33 LCs (VRC01-class^VK1–33^ B cells) was 1 in 583,000 in SE09 mice, which was approximately 3 times higher than the frequency of VRC01-class^VK1–33^ naïve B cells in humans (1 in 1.78 million) (fig. S1C) (16, 44, 45). The LCDR3s from VRC01-class^VK1–33^ naïve B cells for humans and SE09 mice were similar, with slightly less diversity seen in the sequences from SE09 mice (fig. S1D). We expressed BCRs from VRC01-class naïve B cells as IgG mAbs and assessed them for binding to eOD-GT8 using surface plasmon resonance (SPR). VRC01-class naïve mAbs from SE09 mice had nearly identical geomean dissociation constant (K_D_) for eOD-GT8 as previously isolated human naive VRC01-class mAbs (16, 44, 45) (3.5 μM for SE09 [N=42] vs. 3.4 μM for humans [N=73]) (fig. S1E). Restricting the analysis to VRC01-class^VK1–33^ naïve BCRs, the geomean K_D_ for binding to eOD-GT8 was also nearly identical for antibodies from SE09 mice or humans (1.5 μM for SE09 [N=11] vs. 1.6 μM for humans [N=14]) (fig. S1F). Overall, the SE09 mouse provided a model system with a diverse B cell repertoire containing VRC01-class naïve precursors with comparable affinities to human VRC01-class naïve precursors that were present at physiologically relevant frequencies.

### First-boost candidates were designed to boost maturation of VRC01-class BCRs primed by eOD-GT8 60mer.

An ideal first-boost immunogen to follow eOD-GT8 60mer priming would be the most native-like protein that can bind to eOD-GT8-induced VRC01-class BCRs and have no measurable affinity for eOD-GT8-induced non-VRC01-class BCRs specific for the CD4bs. In humans, eOD-GT6 and more native variants of eOD-GT6 have been shown to bind to eOD-GT8-primed VRC01-class BCRs but to have limited binding to eOD-GT8-primed non-VRC01-class BCRs (26). Although eOD-GT6 variants could potentially function as a first boost, immunogens that are more native-like in structure are preferred because they have the potential to shepherd B cells further down the path towards producing bnAbs. Structural platforms that would be more native-like than eOD-GT6 variants would need to include both core-gp120- and trimer-based immunogens, in which trimer-based immunogens would be the closest in structure to the native glycoprotein.

**Fig. 1.**
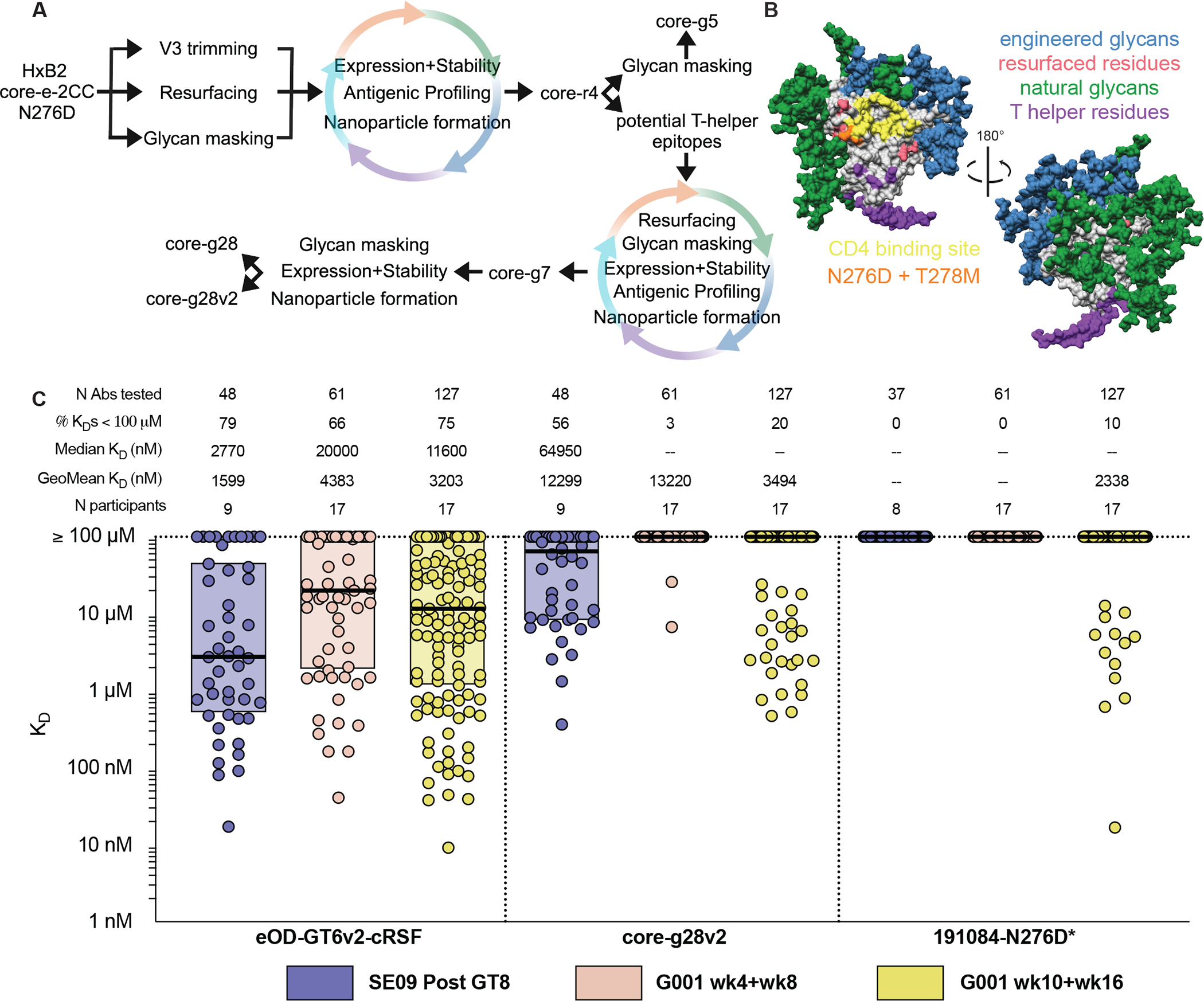
Design and characterization of the core-g28v2 60mer boost immunogen. **(A)** Shown is the iterative immunogen design workflow diagram to improve upon the starting HxB2 core-e-2cc N276D immunogen. **(B)** Shown is a surface representation of a computational model of core-g28v2 monomer colored with CD4bs (yellow), N276D and T278M mutations (orange), resurfaced residues (pink), natural glycosylation sites (green), engineered glycosylation sites (blue), and mutations to add potential CD4^+^ T helper cell epitopes conserved with the HIV env trimer (TH6 residues, purple). **(C)** K_D_ values were measured by SPR for mAbs elicited by eOD-GT8 60mer protein in humans and SE09 mice for first-boost immunogen candidates eOD-GT6v2-cRSF, core-g28v2, and 191084-N276D. Thick lines indicate median values, boxes show 25 and 75% percentile values. *The low-capture IgG SPR method may include some avidity for trimeric analytes.

We set out to design a gp120 core-based nanoparticle immunogen that would boost and drive further maturation of eOD-GT8-primed VRC01-class B cells, but not boost eOD-GT8-primed non-VRC01-class B cells. Starting from a conformationally stabilized HxB2 gp120 core lacking the N276 glycan (HxB2 core-e-2cc N276D) (34), we carried out iterative rounds of remodeling (minimization of the already trimmed V3 to reduce potential immunogenicity of this region), resurfacing (to minimize cross-reactivity to eOD-GT8 outside the CD4bs and to improve affinity for eOD-GT8-induced VRC01-class BCRs) and glycan masking (to reduce responses outside the CD4bs) (Fig. 1A and B). New constructs were screened for protein expression and stability, antigenicity for VRC01-class bnAbs and eOD-GT8-induced VRC01-class Abs from a V_H_1–2 mouse model (22) and from IAVI G001, and nanoparticle formation (Fig. 1A). For some core designs, we extended the N- and C-termini and adjusted two internal segments to increase the content of sequence shared with largely conserved, non-glycosylated regions of HIV Env trimers, as an attempt to provide for potential shared T cell help between our core booster and any subsequent trimer boost. The design path without added potential CD4 helper T cell epitopes resulted in the immunogen core-g5 (Fig. 1A and fig. S2A). The design path with added potential CD4 helper T cell epitopes was subjected to additional rounds of protein resurfacing and glycan masking followed by screening for protein expression and stability, antigenicity, and nanoparticle formation, ultimately resulting in two immunogens, core-g28 and core-g28v2 (Fig. 1A and fig. S2A). All three core designs (core-g5, core-g28, and core-g28v2) were more native-like within the CD4bs than eOD-GT8 (fig. S2B) and assembled into well-formed 60mer nanoparticles (fig. S3A to E). Among these three designs, core-g28v2 60mer was prioritized due to its improved potential for priming CD4 T helper responses that might be engaged by subsequent trimer boosts, superior nanoparticle assembly compared to core-g28 (fig. S3A), lack of affinity for CD4bs-specific non-VRC01-class mAbs induced by eOD-GT8 60mer in G001 (fig. S3F), and favorable affinity gradient, with considerably higher affinity for VRC01-class bnAbs than for eOD-GT8 60mer-induced VRC01-class mAbs (fig. S3G), which should help guide maturation toward bnAb development. Additionally, core-g28v2 lacked detectable affinity for VRC01-class^VK1–33^ precursors isolated from the SE09 mouse (fig. S3H) and is therefore unlikely to prime VRC01-class responses. Glycan occupancy analysis for core-g28v2 60mer (fig. S3I) showed that only 4 of 12 engineered N-linked glycosylation sites had > 50% occupancy, whereas 14 of 16 native N-linked glycosylation sites had > 50% occupancy.

Several additional booster immunogens have been suggested or used in previous studies, including but not limited to: a stabilized native HxB2 gp120 core lacking the N276 glycan (16, 26), a chimeric gp120 core immunogen termed c13 lacking the N276 glycan (19, 22), and a BG505 gp120 core lacking the N276 glycan with additional germline-targeting mutations (18). Finally, a native HIV Env trimer lacking the N276 glycan could theoretically be used as a first-boost immunogen, provided it had appreciable affinity to eOD-GT8-primed VRC01-class BCRs. Therefore, we used SPR to assess affinities of mAbs isolated from humans after one (G001 wk4+wk8) or two (G001 wk10+wk16) immunizations with eOD-GT8 60mer protein (26) and from SE09 mice primed with one dose of eOD-GT8 60mer protein to the following first-boost candidates: eOD-GT6v2-cRSF (a resurfaced version of eOD-GT6v2 (26)), core-g28v2, 191084-N276D stabilized Env trimer (from isolate 191084_B7_19, hereafter abbreviated as 191084), c13.G4.2 core (38), and BG505-core-VRC01-GT3.3 (21) (Fig. 1C, fig. S2 and S4). VRC01-class mAbs isolated in the IAVI G001 trial after eOD-GT8 60mer immunization had affinity for boost immunogen candidates that decreased as the immunogens became more native (Fig. 1C, fig. S2 and S4). Overall, core-g28v2 showed moderate affinity for eOD-GT8 elicited mAbs (Fig. 1C), could assemble into a high avidity 60mer nanoparticle (fig. S3), and contained a more native CD4bs than eOD-GT6v2-cRSF and the other gp120 core immunogens (fig. S2).

### VRC01-class B cells were boosted with protein immunogens.

We evaluated core-g28v2 60mer and six other candidate first-boost immunogens as adjuvanted proteins in SE09 mice (table S1). SE09 mice were primed with eOD-GT8 60mer protein, received first-boost immunogens at week 6, and responses were evaluated at weeks 6 and 12. The primary readouts were the frequencies, degrees of mutation, and affinities of VRC01-class responses. Splenocytes and draining lymph node cells from week 6 (post-prime) or week 12 (post-boost) were sorted for antigen-specific CD19^+^/IgM^-^IgD^-^ B cells and subjected to paired single-cell BCR sequencing using 10x Genomics followed by bioinformatic analyses (fig. S5). Samples from each group were sorted with antigens matched to the immunogen received; placebo samples were sorted with core-g28v2. We refer to the sorted cells as antigen-specific memory B cells (MBCs), because they were expected to be enriched in memory-phenotype cells, although other types of cells such as germinal center B cells could also be present.

**Fig. 2.**
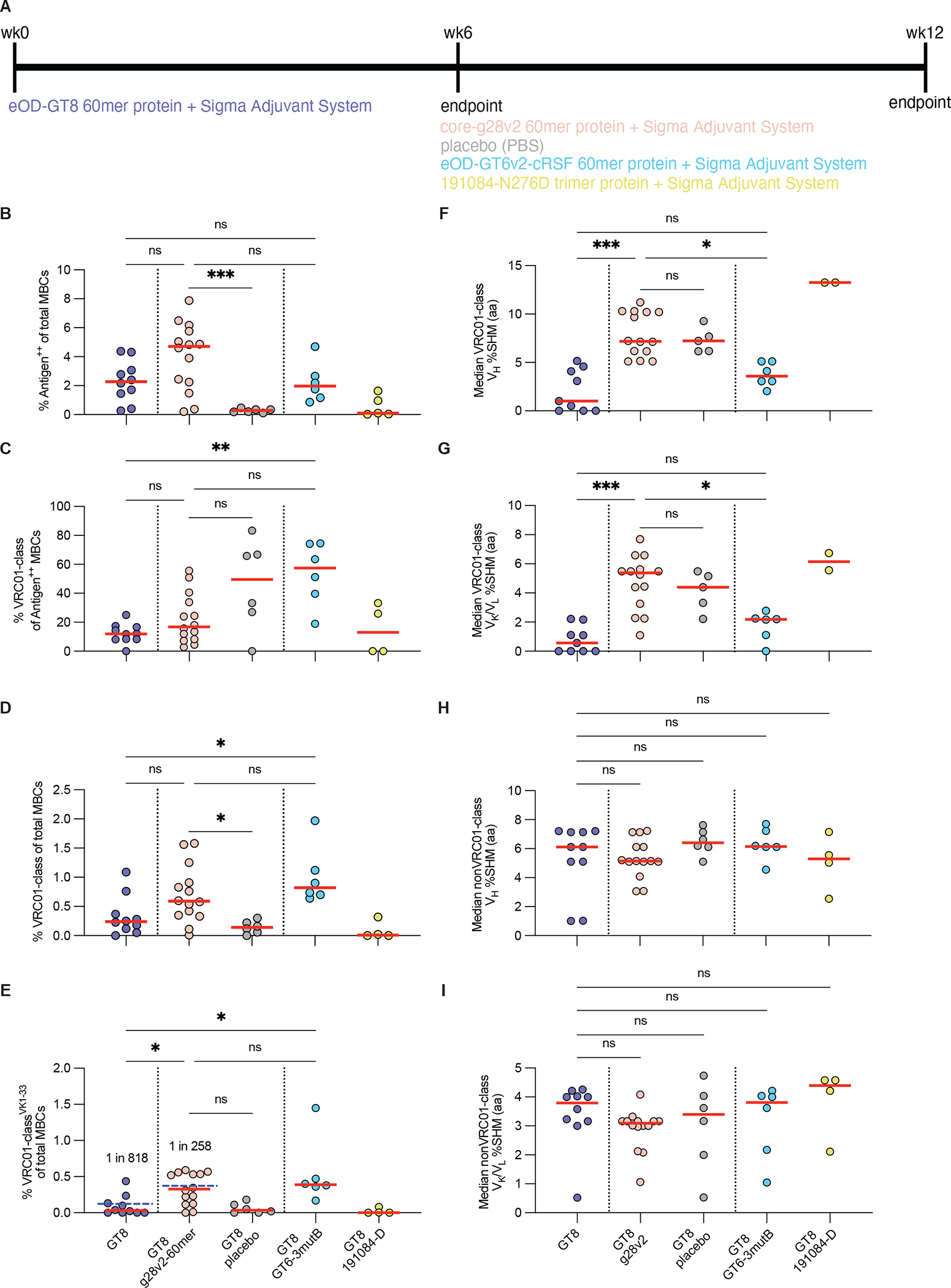
Comparison of protein boost immunogens. **(A)** Shown is the immunization scheme for evaluating boost immunogen candidates delivered as proteins plus adjuvant in SE09 mice. **(B)** The frequency of antigen^++^ MBCs among total MBCs was analyzed by flow cytometry. Each group was sorted with matched antigens, except for the PBS placebo group which was sorted with the core-g28v2 probe. **(C to E)** Shown is the frequency of VRC01-class MBCs among antigen^++^ MBCs (C), the frequency of VRC01-class MBCs among total MBCs (D), and the frequency of VRC01-class MBCs with human V_K_1–33 light chains among total MBCs (E). The blue dashed bar in (E) indicates the overall frequency of VRC01-class MBCs with human V_K_1–33 light chains among total MBCs within each group. Red bars indicate medians and each point represents an individual mouse for (B to E). **(F** and **G)** The median percent aa SHM in the V_H_ gene (F) and in the V_K_/V_L_ genes (G) is shown for all VRC01-class MBCs. **(H** and **I)** The median percent aa SHM in the V_H_ gene (H) and in the V_K_/V_L_ genes (I) is shown for non-VRC01-class MBCs. Each point represents the median per mouse and the red bars indicate the median of medians for panels (F to I). Statistical comparisons were made by Kruskal-Wallis test followed by Dunn’s test for multiple comparisons. *p<0.05, **p<0.01, ***p<0.001; ns, not significant.

In the primary set of experiments, mice were primed with eOD-GT8 60mer protein and then boosted with either core-g28v2 60mer, eOD-GT6v2-cRSF 60mer, 191084-N276D soluble native Env trimer, or phosphate buffered saline (PBS) as a placebo (Fig. 2A). Antigen-specific MBCs were detected in all immunization groups, although frequencies were very low for both the placebo and 191084-N276D groups (Fig. 2B). Substantial fractions of the antigen-specific MBCs were VRC01-class for both the core-g28v2 and eOD-GT6v2-cRSF boost groups (Fig. 2C), and median frequencies of VRC01-class MBCs were 0.5% to 1% for those two boost groups (Fig. 2D). In contrast, VRC01-class MBCs were detected in only two of the five animals boosted with the 191084-N276D trimer (Fig. 2C), and an appreciable frequency of VRC01-class MBCs was detected in only one such animal (Fig. 2D), from which we concluded that this particular N276-lacking native-like soluble trimer would not be an effective first boost.

Consideration of VRC01-class^VK1–33^ responses offered the most direct comparison to human responses. Priming with eOD-GT8 60mer protein led to a 712-fold expansion of VRC01-class^VK1–33^ MBCs relative to the naïve IgD^+^IgM^+^ VRC01-class^VK1–33^ precursor pool in SE09 mice (Fig. 2E and fig. S1C). For comparison, in the G001 clinical trial, the frequency of VRC01-class^VK1–33^ IgG^+^ B cells was 45-fold or 317-fold higher than the naive VRC01-class^VK1–33^ precursor frequency at eight weeks after the first or second dose of eOD-GT8 60mers, respectively (26). Thus, a single immunization with adjuvanted eOD-GT8 60mer protein in SE09 mice was approximately as effective as two immunizations in humans for inducing substantial frequencies of VRC01-class^VK1–33^ MBCs.

Boosting with core-g28v2 60mer after one immunization of eOD-GT8 60mer in SE09 mice led to an additional 3-fold expansion of VRC01-class^VK1–33^ MBCs, resulting in a VRC01-class^VK1–33^ frequency of 1 in 258 MBCs at week 12 (Fig. 2E). Compared to a placebo boost, the core-g28v2 60mer boost elicited higher frequencies of antigen-specific MBCs (median 4.7% vs. 0.3%, p=0.009; Fig. 2B) and VRC01-class MBCs (median 0.59% vs. 0.14%, p=0.032; Fig. 2D). Furthermore, core-g28v2 lacked detectable affinity for VRC01-class^VK1–33^ precursors in the SE09 mouse (fig. S3H), hence it was not expected to elicit de novo VRC01-class responses. The increased VRC01-class frequencies detected after the core-g28v2 60mer boost were therefore most likely due to boost-induced expansion of eOD-GT8 60mer-primed B cells and not to either de novo priming of VRC01-class responses by the boost immunogen or to evolution of the eOD-GT8 60mer-primed cells over time independent of the boost.

Boosting with eOD-GT6v2-cRSF 60mer elicited similar frequencies of VRC01-class MBCs and VRC01-class^VK1–33^ MBCs compared to core-g28v2 60mer (Fig. 2D and E). However, V_H_ and V_K_/V_L_ somatic hypermutation (SHM) among VRC01-class MBCs did not increase after boosting with eOD-GT6v2-cRSF 60mer relative to the post-prime response (Fig. 2F and G). In contrast, boosting with core-g28v2 60mer produced VRC01-class MBCs with higher V_H_ and V_K_/V_L_ SHM compared to the post-prime response and compared to boosting with eOD-GT6v2-cRSF 60mer (Fig. 2F and G). As the core-g28v2 60mer boost generated similar VRC01-class MBC frequencies but higher SHM in VRC01-class MBCs (and similar SHM in non-VRC01-class MBCs) compared to the eOD-GT6v2-cRSF 60mer boost, and as core-g28v2 had a more native-like structure than eOD-GT6v2-cRSF (fig. S2), we concluded that core-g28v2 60mer was superior to eOD-GT6v2-cRSF 60mer as a first-boost immunogen candidate.

**Fig. 3.**
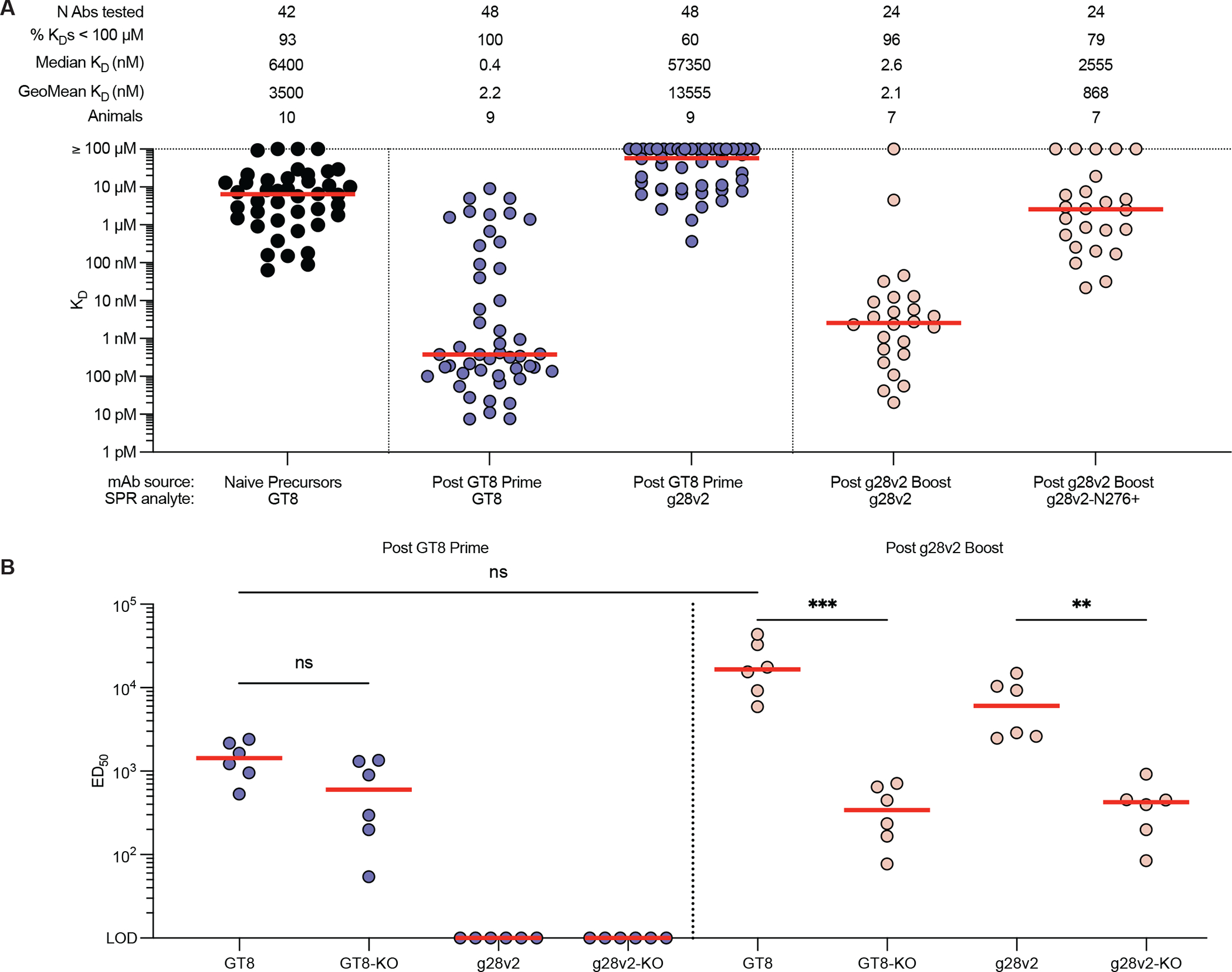
mAb SPR and serum antibody binding responses after eOD-GT8 60mer priming and core-g28v2 boosting. **(A)** Monovalent K_D_ values were measured by SPR of VRC01-class mAbs from naïve SE09 mice, eOD-GT8 60mer primed sE09 mice, and eOD-GT8 60mer primed, core-g28v2 60mer boosted SE09 mice for eOD-GT8, core-g28v2, and core-g28v2-N276^+^. Red bars indicate median affinities and include non-binders. Geomean affinities were calculated among binders only. **(B)** Shown is serum IgG ELISA binding to eOD-GT8, eOD-GT8-KO, core-g28v2, and core-g28v2-KO for SE09 mice primed with eOD-GT8 60mer protein (purple) alone or SE09 mice primed with eOD-GT8 60mer protein and boosted with core-g28v2 60mer protein (pink). Each point represents the half-maximal effective dilution (ED_50_) of serum per mouse. Red bars indicate median ED_50_ values. Statistical comparisons were made by Kruskal-Wallis test followed by Dunn’s test for multiple comparisons. **p<0.01, ***p<0.001; ns, not significant.

BCR sequences from the eOD-GT8 60mer prime and core-g28v2 60mer boost immunization groups were expressed as mAbs and assessed for affinity to eOD-GT8, core-g28v2, and core-g28v2 with the N276 glycan (Fig. 3A). The geomean affinity of post-prime VRC01-class mAbs for eOD-GT8 increased by over 1000-fold relative to the affinity of the VRC01-class naïve precursors from SE09 mice (Fig. 3A). Approximately half of the post-prime VRC01-class mAbs assayed had measurable affinity for core-g28v2, with geomean affinity among binders of approximately 12 μM (Fig. 3A). Boosting with core-g28v2 60mer led to a 5900-fold increase in geomean affinity for core-g28v2 relative to the post-prime VRC01-class mAbs and produced VRC01-class mAbs that were able to bind a variant of core-g28v2 containing the N276 glycan (Fig. 3A).

We used serum enzyme-linked immunosorbent assay (ELISA) analyses to understand the degree to which serum antibodies primed by eOD-GT8 60mer might interfere with core-g28v2 60mer boosting. We found that serum antibody responses post eOD-GT8 60mer prime bound to both eOD-GT8 and the epitope knockout version of eOD-GT8, but showed no detectable binding to core-g28v2 or the epitope knockout version of core-g28v2, suggesting that post-prime serum antibodies would not contribute to antibody feedback during the boosting immunization (Fig. 3B). Boosting with core-g28v2 60mer elicited serum antibodies with higher 50% effective dilution (ED_50_) values for eOD-GT8 and core-g28v2 relative to the corresponding epitope-knockout versions, indicating that the core-g28v2 boost induced a strong CD4bs-specific response (Fig. 3B), as intended by the core-g28v2 design.

To evaluate other alternatives to core-g28v2 60mer, we also tested boosting with core-g28v2 as a 24mer ferritin nanoparticle, c13.G4.2 as a 24mer ferritin nanoparticle (38) or a 60mer nanoparticle, and BG505-core-VRC01-GT3.3 60mer (21) (fig. S6). None of the alternative booster immunogens were superior to core-g28v2 60mer in terms of the magnitude of the VRC01-class MBC response or the amount of SHM induced (fig. S6). In addition, the amounts of SHM among boost-antigen-specific, non-VRC01-class BCRs were similar for the different boosters (fig. S6H and I) and not different from the SHM following priming with eOD-GT8 60mer (fig. 2H and I), suggesting that each first-boost immunogen elicited a de novo non-VRC01-class response, but none caused substantial boosting of eOD-GT8 60mer-primed non-VRC01-class B cells. From these analyses, we selected core-g28v2 60mer as our lead first-boost candidate to follow eOD-GT8 60mer priming.

### The number of key VRC01-class residues was increased by protein and adjuvant boosting.

**Fig. 4.**
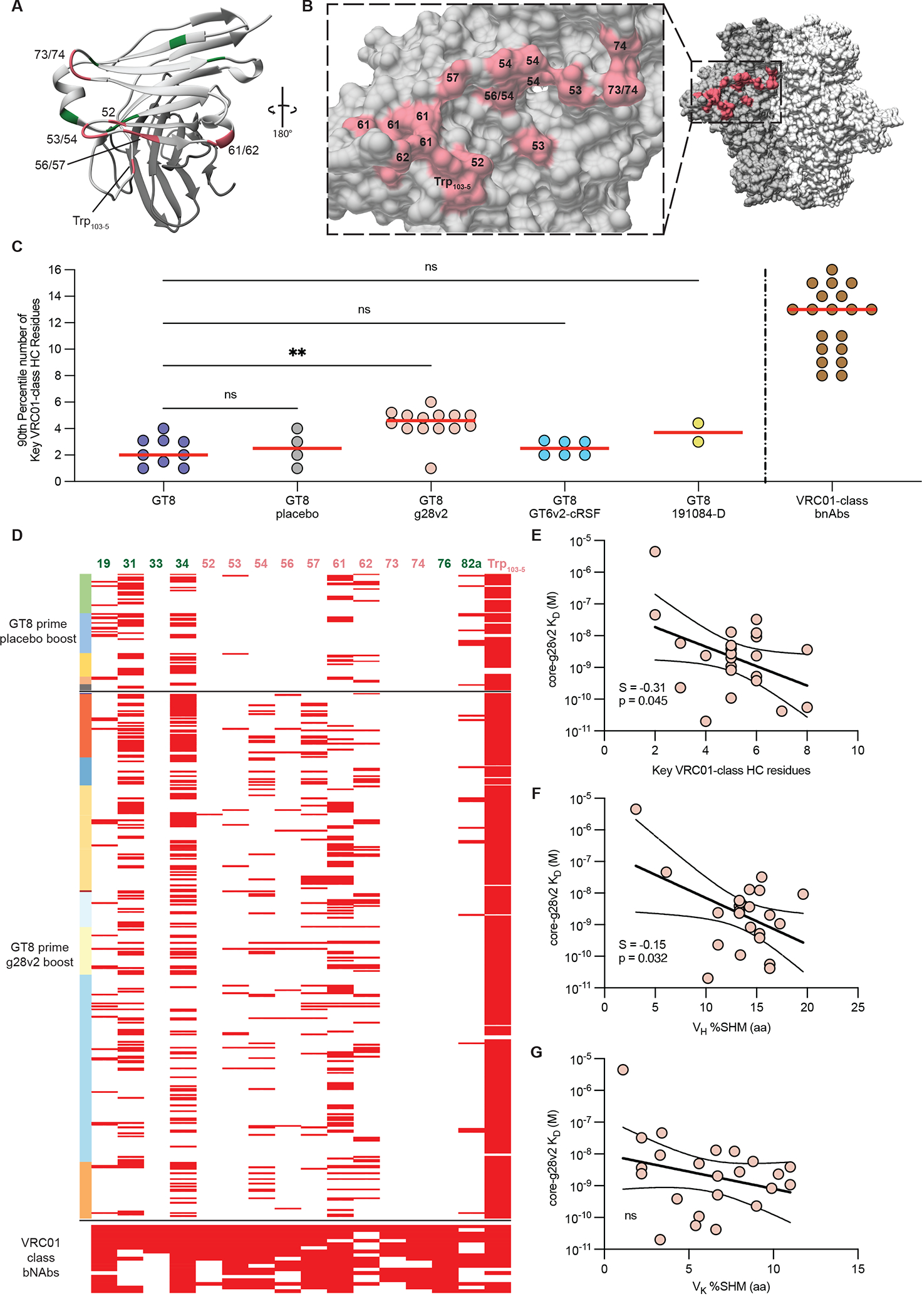
Key VRC01-class heavy chain residues after protein immunization. **(A)** Shown is a ribbon diagram of the VRC01 variable fragment (Fv) with key VRC01-class heavy chain residues colored green for non-paratope residues and pink for paratope residues. **(B)** A molecular surface representation of HIV Env shows contact residues of key VRC01-class heavy chain paratope residues (red). **(C)** Shown is the 90^th^ percentile number of key VRC01-class heavy chain residues elicited during immunization experiment described in Fig. 2A. Each point is the 90^th^ percentile number of key VRC01-class heavy chain residues for each mouse with the red bars indicating the median of the 90^th^ percentile values for each group. Statistical comparisons were made by Kruskal-Wallis test followed by Dunn’s test for multiple comparisons. **p<0.01; ns, not significant. **(D)** Shown are key VRC01-class heavy chain residues for all VRC01-class sequences recovered for placebo and core-g28v2 boost groups. Numbers at top indicate positions within the antibody, colored as in (A); column at left indicates data from each mouse in a different color; each row indicates a single VRC01-class sequence, with red boxes indicating the presence of non-germline key VRC01-class heavy chain residues. Residue numbers in panels (A), (B), and (D) use the Kabat antibody numbering scheme (56). **(E to G)** K_D_ values between core-g28v2 and mAbs isolated after core-g28v2 60mer boosting were correlated with number of key VRC01-class heavy chain residues (E), percent V_H_ SHM (F), and percent V_K_ SHM (G). In (E to G), solid lines show correlations, dashed lines show 95% confidence internal, S represents slope, and p represent the P-value from a simple linear regression test. ns, not significant.

We selected a representative panel of 19 VRC01-class bnAbs with minimal (≤ 3 aa) indels as aspirational goals for vaccine elicitation (26) and performed sequence analysis to identify key residues commonly utilized by VRC01-class bnAbs. As described previously (26), we identified 20 positions (19 within V_H_1–2, plus Trp_103–5_) as key VRC01-class residues, four of which are germline-encoded in the V_H_1–2*02 and *04 alleles (47W, 50W, 55G, and 71R). We counted the key VRC01-class residues on a scale ranging from −4 to +16, to allow for all possibilities from losing all germline-encoded key residues, to gaining key residues at all 16 non-germline-encoded positions (26). Here, structural analysis of the 16 non-germline-encoded key VRC01-class residues revealed that 10 residues were involved in the VRC01-class paratope (Fig. 4A) and made contact with the CD4bs and adjacent regions on HIV Env (Fig. 4B). The six non-paratope key VRC01-class residues likely stabilize the HCDR2 of VRC01-class bnAbs or potentially make contact with distal N-linked glycans.

To assess the ability of different prime-boost regimens to drive maturation toward bnAb development, we counted the number of key VRC01-class residues in each VRC01-class BCR isolated for each animal, then computed the 90^th^ percentile number of key VRC01-class residues per animal (as representative of the best 20% of BCRs per animal), and finally computed the median 90^th^ percentile number of key VRC01-class residues for each immunization group (26). We carried out this analysis for different groups primed with eOD-GT8 60mer and boosted with core-g28v2 60mer, eOD-GT6v2-cRSF 60mer, 191084-N276D trimer, or PBS placebo (Fig. 4C). The median 90^th^ percentile number of key VRC01-class residues increased significantly from approximately +2 after priming with eOD-GT8 60mer to approximately +5 after boosting with core-g28v2 60mer (p=0.002) (Fig. 4C). None of the other boosts resulted in an increase in key VRC01-class residues relative to post-prime (Fig. 4C). For comparison, the representative VRC01-class bnAbs had a median of 13 key residues, and a range of +8 to +16 (Fig. 4C and fig. S7A), illustrating that additional maturation will be required to elicit bnAbs. Paratope key VRC01-class residues at positions within the HCDR2 (54, 56, and 57) were detected after boosting with core-g28v2 60mer, but not after priming with eOD-GT8 60mer, whether followed by a placebo boost (Fig. 4D) or not (fig. S7B), illustrating a direct effect of the boost. Key paratope VRC01-class residues at positions within the frame work region 3 (FWR3; 73 and 74) make contact with residues on HIV Env that are either absent on core-g28v2 or are present in a different conformation (Fig. 4B). Accordingly, key VRC01-class residues at positions 73 and 74 were not detected after boosting with core-g28v2 60mer and would likely need to be elicited using a native HIV Env trimer (Fig. 4D). The number of key VRC01-class residues and the V_H_ amino acid (aa) SHM both correlated with higher affinity binding to core-g28v2 (Fig. 4E and F), indicating that in vivo selection for higher affinity to core-g28v2 served to guide immune responses toward bnAb development. eOD-GT8 and core-g28v2 make little contacts with the light chains of VRC01-class antibodies, except for the LCDR3. Hence it was not surprising that the V_K/L_ aa SHM was not correlated with higher affinity binding to core-g28v2 (Fig. 4G). We concluded that the core-g28v2 60mer boost immunization selected for desirable maturation of VRC01-class responses.

### eOD-GT8 60mer mRNA primes VRC01-class responses.

**Fig. 5.**
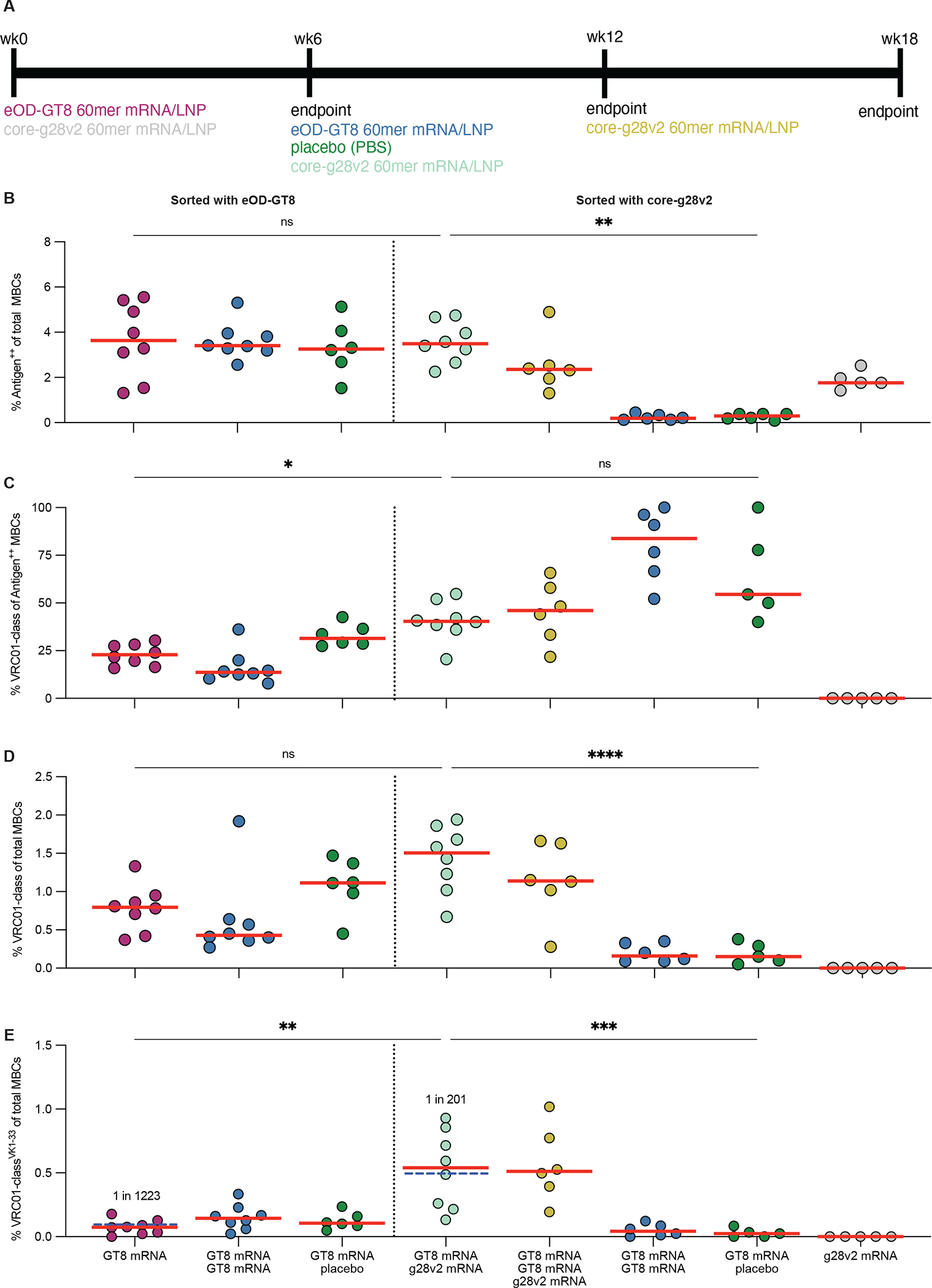
Immune response elicited using mRNA/LNP immunization. **(A)** Shown is the immunization scheme for evaluating immunogens delivered using mRNA/LNPs in SE09 mice. **(B)** The frequency of antigen^++^ MBCs among total MBCs was analyzed by flow cytometry. **(C to E)** Shown is the frequency of VRC01-class MBCs among antigen^++^ MBCs (C), the frequency of VRC01-class MBCs among total MBCs (D), and the frequency of VRC01-class MBCs with human V_K_1–33 light chains among total MBCs (E). Red bars indicate medians and each point represents an individual mouse for panels (B to E). Statistical comparisons were made by Kruskal-Wallis test followed by Dunn’s test for multiple comparisons. *p<0.05, **p<0.01, ***p<0.001, ****p<0.0001; ns, not significant.

**Fig. 6.**
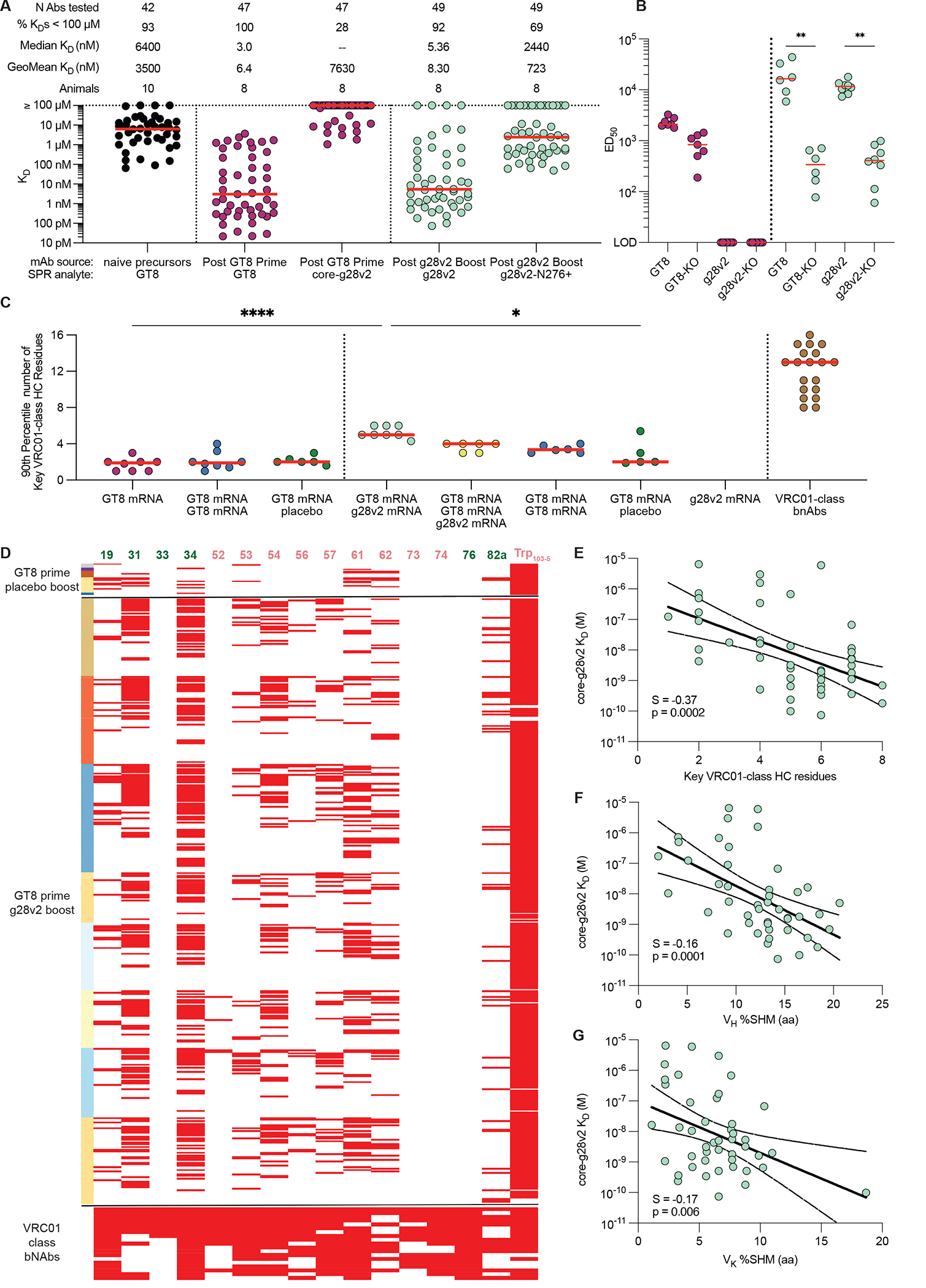
Key VRC01-class heavy chain residues, mAb SPR, and serum antibody binding after mRNA/LNP immunization. **(A)** Shown are monovalent K_D_ values measured by SPR of VRC01-class mAbs from naïve SE09 mice, eOD-GT8 60mer mRNA/LNP primed SE09 mice, and eOD-GT8 60mer mRNA/LNP primed, core-g28v2 60mer mRNA/LNP boosted SE09 mice for eOD-GT8, core-g28v2, and core-g28v2-N276^+^. Red bars indicate median affinities and include non-binders. Geomean affinities were calculated among binders only. **(B)** Shown is serum IgG ELISA binding to eOD-GT8, eOD-GT8-KO, core-g28v2, and core-g28v2-KO for SE09 mice primed with eOD-GT8 60mer mRNA/LNPs (magenta) only or primed and boosted with core-g28v2 60mer mRNA/LNPs (green). Each point represents the ED_50_ value of serum per mouse. Red bars indicate median ED_50_ values. **(C)** Shown are the 90^th^ percentile values for key VRC01-class heavy chain residues elicited during mRNA/LNP immunization experiment described in Fig. 5A. Each point is the 90^th^ percentile key VRC01-class heavy chain residues for each mouse with the red bars indicating the median of the 90^th^ percentile values for each group. Statistical comparisons in (B and C) were made by Kruskal-Wallis test followed by Dunn’s test for multiple comparisons. *p<0.05, **p<0.01, ****p<0.0001; ns, not significant. (**D)** Key VRC01-class heavy chain residues for all VRC01-class sequences recovered for placebo and core-g28v2 boost groups. Numbers at top indicate positions within the antibody, colored as in (Fig. 4A); column at left indicates data from each mouse in a different color; each row indicates a single VRC01-class sequence, with red boxes indicating the presence of non-germline key VRC01-class heavy chain residues. Residue numbers use the Kabat antibody numbering scheme (56). **(E to G)** K_D_ values between core-g28v2 and mAbs isolated after core-g28v2 60mer mRNA/LNP boosting were correlated with number of key VRC01-class heavy chain residues (E), percent V_H_ SHM (F), and percent V_K_ SHM (G). In (E to G), solid lines show correlations, dashed lines show 95% confidence internal, S represents slope, and p represent the P-value from a simple linear regression test.

mRNA/LNP vaccine platforms can provide excellent immunogenicity and safety with rapid timelines for entering clinical trials, as illustrated by the SARS-CoV-2 mRNA vaccines (46, 47). Using Moderna mRNA/LNP immunogens in the SE09 mouse model, we evaluated eOD-GT8 60mer priming (one or two immunizations) and core-g28v2 60mer boosting (after one or two eOD-GT8 60mer priming immunizations), and as controls we tested a placebo boost and a core-g28v2 60mer priming group (Fig. 5A). B cell analyses were carried out as for the protein experiments, except that replicate samples from placebo-boosted animals were sorted twice, once with eOD-GT8 probes and once with core-g28v2 probes. Similar frequencies of antigen-specific (eOD-GT8-specific) MBCs were induced by one or two immunizations with eOD-GT8 60mer mRNA or by eOD-GT8 60mer mRNA followed by placebo (Fig. 5B), suggesting that the second immunization of eOD-GT8 60mer mRNA had little effect. Furthermore, the percentage of eOD-GT8-specific MBCs that were VRC01-class was not significantly different for one eOD-GT8 60mer immunization followed by placebo compared to two immunizations with eOD-GT8 60mer (Fig. 5C), indicating that the second eOD-GT8 60mer mRNA immunization did not cause substantial additional priming of VRC01-class precursors. One or two eOD-GT8 60mer mRNA immunizations induced similar frequencies of VRC01-class and VRC01-class^VK1–33^ MBCs (Fig. 5D and E), and the VRC01-class MBCs in both cases had minimal V_H_ and V_K/L_ SHM (fig. S8A and B). Comparing mRNA to adjuvanted protein for one eOD-GT8 60mer immunization, we found that mRNA vaccination induced higher frequencies of VRC01-class MBCs (median values of 0.8% vs. 0.24%, *P*=0.0067, fig. S9A), similar frequencies of VRC01-class^VK1–33^ MBCs (median values of 0.07% vs. 0.03%, *P*=0.76, fig. S9B), low SHM in both cases (e.g. median V_H_ SHM values of 0% vs. 1%, *P*=0.07, fig. S9C), and similar numbers of key VRC01-class residues (median 90^th^ percentile values of 1.9 vs 2, *P*=0.23, fig. S9D). In terms of VRC01-class mAbs affinities, the results for mRNA were also similar to the case of protein immunization: priming with eOD-GT8 60mer mRNA resulted in a >500-fold increase in the geomean affinity of VRC01-class mAbs for eOD-GT8 relative to the affinity of the VRC01-class naïve precursors from SE09 mice (Fig. 6A), with similar geomean K_D_ values of 6 nM and 2 nM from mRNA and protein immunization, respectively. Serum antibody responses post eOD-GT8 60mer mRNA prime bound to both eOD-GT8 and the epitope KO version of eOD-GT8, but showed no detectable binding to core-g28v2 or the epitope KO of core-g28v2 (Fig. 6B), analogous to what we found with the protein immunizations (Fig. 3B). We concluded that mRNA performed as well or better than adjuvanted protein for eOD-GT8 60mer priming in the SE09 mouse, and that two immunizations was not better than one.

### Core-g28v2 60mer mRNA/LNP boosting enhances VRC01-class responses.

Having dissected the frequency and SHM effects of eOD-GT8 60mer mRNA priming, we then turned our attention to analysis of core-g28v2 60mer mRNA boosting experiments and controls. After one eOD-GT8 60mer mRNA priming immunization, boosting with core-g28v2 60mer mRNA elicited higher frequencies of core-g28v2-specific MBCs, VRC01-class MBCs, and VRC01-class^VK1–33^ MBCs compared to placebo boost (Fig. 5B, D, and E). Furthermore, priming with core-g28v2 60mer mRNA elicited antigen-specific MBCs, but no detectable VRC01-class MBCs (Fig. 5B and C), confirming that the increased frequency of VRC01-class MBCs detected after core-g28v2 60mer mRNA boosting was due to expansion of eOD-GT8 60mer-primed VRC01-class B cells and not to de novo VRC01-class priming by core-g28v2 60mer. In concert with that expansion, heterologous boosting induced BCR maturation: boosting with core-g28v2 60mer mRNA after a single eOD-GT8 60mer mRNA prime elicited higher amounts of V_H_ SHM among VRC01-class MBCs compared to boosting with placebo (median SHM values of 8.7% vs. 6.1% for V_H_ [*P*=0.02]) (fig. S8A and B, with core-g28v2 and placebo boost groups sorted at week 12 with core-g28v2 probes). Boosting with core-g28v2 60mer mRNA after a single eOD-GT8 60mer mRNA prime also elicited higher amounts of key VRC01-class HC residues among VRC01-class MBCs compared to boosting with placebo (median of 90^th^ percentile values of 5 vs. 2 for core-g28v2 and placebo boost groups sorted at week 12 with core-g28v2 probes, respectively, *P*=0.02; Fig. 6C). After eOD-GT8 60mer mRNA priming, VRC01-class^VK1–33^ BCRs were enriched for LCDR3s with Glu at position 96, the most prevalent residue at that position in VRC01-class^VK1–33^ bnAbs (fig. S10A versus fig. S1D), and this enrichment increased after core-g28v2 60mer mRNA boosting (fig. S10B). Comparing the output of the core-g28v2 60mer mRNA boost after one or two priming immunizations with eOD-GT8 60mer mRNA, we detected no improvements in frequencies of antigen-specific MBCs, VRC01-class MBCs, or VRC01-class^VK1–33^ MBCs associated with double priming (Fig. 5B to E), and we found that the double priming regimen induced significantly lower amounts of V_H_ and V_K/L_ SHM (p<0.001 and p<0.05 respectively) (fig. S8A and B) and key VRC01-class residues (p<0.001) (Fig. 6C). Hence, in the SE09 mouse, the core-g28v2 60mer mRNA boost was effective for expanding and maturing VRC01-class responses after a single eOD-GT8 60mer mRNA prime, and there was no benefit to using a double eOD-GT8 60mer mRNA prime.

We used multiple metrics to compare the performance mRNA to adjuvanted protein for one eOD-GT8 60mer immunization followed by a core-g28v2 60mer boost. mRNA induced higher frequencies of VRC01-class MBCs (median values of 1.5%% vs. 0.6%, *P*=0.003; fig. S11A), similar frequencies of VRC01-class^VK1–33^ MBCs (median values of 0.33% vs. 0.54%; fig. S11B), similar amounts of SHM (e.g. median V_H_ SHM values of 8.7% vs. 7.2%; fig. S11C), and similar numbers of key VRC01-class HC residues (median 90^th^ percentile values of 5 vs. 4.6; fig. S11D). In terms of mAb affinity analysis, mRNA performed similarly as protein. Approximately one third of the post-prime VRC01-class mAbs assayed had measurable affinity for core-g28v2, with a geomean K_D_ among binders of about 7.6 μM (Fig. 6A), values similar to those obtained with protein immunization (Fig. 3A). Boosting with core-g28v2 60mer mRNA led to a 900-fold increase in geomean affinity for core-g28v2 relative to the post-prime VRC01-class mAbs and produced VRC01-class mAbs that were able to bind a more native-like variant of core-g28v2 containing the N276 glycan (Fig. 6A), with a geomean K_D_ among binders of 720 nM similar to the value of 870 nM obtained for protein immunization (Fig. 3A). Boosting with core-g28v2 60mer mRNA after a single eOD-GT8 60mer mRNA prime induced more key VRC01-class HCDR2 residues compared to priming alone (fig. S12A) or priming followed by a placebo boost (Fig. 6D and fig. S12B), as was true for protein immunization. We also found that boosting with core-g28v2 60mer mRNA after a single eOD-GT8 60mer mRNA prime induced more key VRC01-class HCDR2 residues compared to two immunizations with eOD-GT8 60mer mRNA (fig. S12C and D). The number of key VRC01-class residues, percent V_H_ SHM, and percent V_K_/V_L_ SHM were all correlated with VRC01-class mAb K_D_ for core-g28v2, for mAbs derived from MBCs isolated after core-g28v2 60mer mRNA boosting (Fig. 6E to G), similar to our correlation findings for protein immunization (Fig. 4E to G). To confirm the epitope specificity of the VRC01-class mAbs derived from MBCs isolated after core-g28v2 60mer mRNA boosting, we measured binding to the epitope KO version of core-g28v2 with SPR and assessed competition with VRC01 using biolayer interferometry (fig. S13A and B). Only one VRC01-class mAb had measurable affinity for the epitope KO version of core-g28v2 and it had >8000-fold reduction in affinity relative to the non-epitope KO version of core-g28v2 (fig. S13A) indicating that all of the VRC01-class mAbs targeted the CD4bs epitope. Competition biolayer interferometry (BLI) with VRC01 Fab showed similar results in that mature VRC01 Fab fully blocked binding to all of the VRC01-class mAbs (fig. S13B). In serum antibody binding analyses, the core-g28v2 60mer mRNA boost induced a similar pattern of responses as protein, with significantly higher ED_50_ values for eOD-GT8 and core-g28v2 compared to the epitope KO versions (p<0.001), indicating that the majority of the response elicited after boosting with mRNA was CD4bs-specific (Fig. 6B), as intended by the core-g28v2 design. Furthermore, there were significantly higher ED_50_ values for eOD-GT8 after boosting with core-g28v2 60mer mRNA compared to post eOD-GT8 60mer prime (p<0.001) but no increase in ED_50_ values for eOD-GT8 KO (Fig. 6B), which also suggested that boosting with core-g28v2 60mer preferentially boosted CD4bs-specific responses over non-CD4bs off-target responses. We concluded that mRNA delivery of eOD-GT8 60mer followed by core-g28v2 60mer was effective for priming VRC01-class naive B cells and driving their expansion and early maturation in the SE09 mouse. Similar findings were made in a different stringent mouse model (48), demonstrating the robustness of our conclusions.

### Non-VRC01-class BCR affinity and specificity

Given that our B cell sorting strategy isolated antigen-specific, rather than epitope-specific B cells, it was of interest to determine what proportion of the antigen-specific non-VRC01-class MBCs were CD4bs-specific and therefore direct epitope competitors for the VRC01-class responses. We employed SPR analyses to interrogate the specificity of the non-VRC01-class mAbs recovered from MBCs after eOD-GT8 60mer mRNA priming and core-g28v2 60mer mRNA boosting. Twelve randomly selected non-VRC01-class, eOD-GT8-binding BCRs isolated after priming with eOD-GT8 60mer mRNA were expressed as mAbs and assessed for affinity to eOD-GT8, eOD-GT8-KO, and core-g28v2 (fig. S14A). Half of the mAbs had >50-fold reduction in affinity for eOD-GT8-KO compared to eOD-GT8, indicating that they were CD4bs-specific competitors. None of the mAbs had measurable affinity to core-g28v2 (fig. S14A), suggesting that eOD-GT8 60mer-primed non-VRC01-class MBCs were not readily boosted by core-g28v2. Seventeen randomly selected non-VRC01-class, core-g28v2-binding BCRs isolated after boosting with core-g28v2 60mer mRNA were expressed as mAbs and assessed for affinity to core-g28v2 and core-g28v2 KO (fig. S14B). More than fifty percent of post core-g28v2 non-VRC01-class mAbs had >50-fold reduction in affinity for g28v2-KO compared to core-g28v2, indicating that they too were CD4bs-specific competitors (fig. S14B). We concluded that the expansion and favorable maturation of VRC01-class responses induced by priming with eOD-GT8 60mer mRNA and boosting by core-g28v2 60mer mRNA were achieved in the presence of CD4bs-specific non-VRC01-class competition.

With the design of core-g28v2 aiming to avoid boosting non-VRC01-class responses primed by eOD-GT8, we sought to determine if that goal was achieved. The strongest line of evidence came from SHM analyses in protein vaccination: SHM in VRC01-class MBCs was higher after the core-g28v2 boost compared to post-prime (Fig. 2F and G), but SHM in non-VRC01-class MBCs was similar after the core-g28v2 boost and after the eOD-GT8 prime (Fig. 2H and I). Additionally, the quantities of SHM (V_H_ and V_K_/V_L_) among non-VRC01-class MBCs were comparable between animals primed with core-g28v2 mRNA and those primed with eOD-GT8 mRNA and boosted with core-g28v2 mRNA (fig. S15A and B). These SHM analyses suggested that the core-g28v2-specific, non-VRC01-class responses detected after the core-g28v2 60mer boost were likely de novo primed by the core immunization. In further support of that hypothesis, we found that none of the 12 post-prime non-VRC01-class mAbs tested had detectable affinity for core-g28v2 (fig. S14A).

### Identifying potential boost-2 immunogens

**Fig. 7.**
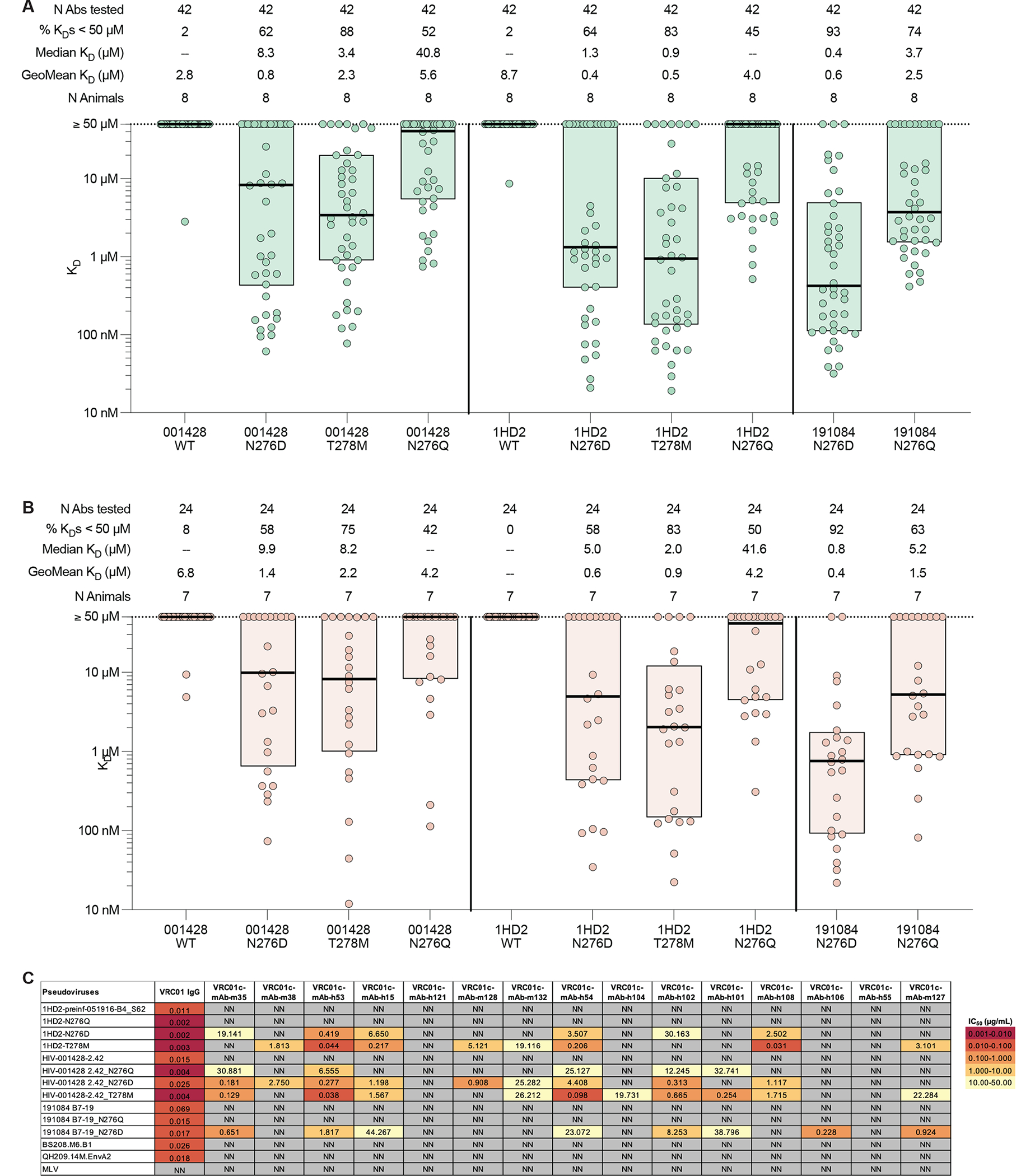
Post core-g28v2 mAbs bind N276(–) native-like HIV Env trimers and neutralize corresponding pseudoviruses. **(A)** Shown are apparent K_D_ values measured by SPR of VRC01-class mAbs elicited in SE09 mice after priming with eOD-GT8 60mer mRNA/LNPs and boosting with core-g28v2 mRNA/LNPs, for wildtype and N276(–) native-like HIV Env trimers. **(B)** Shown are apparent K_D_ values measured by SPR of VRC01-class mAbs elicited in SE09 mice after priming with eOD-GT8 60mer protein + adjuvant and boosting with core-g28v2 60mer protein + adjuvant, for wildtype and N276(–) native-like HIV Env trimers. Thick lines indicate median values, boxes show 25 and 75% percentile values (A and B). **(C)** Pseudoviruses neutralization was tested using a panel of 15 VRC01-class mAbs elicited in SE09 mice after eOD-GT8 60mer mRNA/LNP priming and core-g28v2 60mer mRNA/LNP boosting. Murine leukemia virus (MLV) was used as a negative control. No neutralization: NN; IC_50_ > 50 μg/mL.

We hypothesized that the next step in sequential boosting after core-g28v2 60mer would be to immunize with a prefusion conformation-stabilized HIV Env trimer lacking the N276 glycan (16, 21–23, 38, 49). Therefore, determining if post-core-g28v2 VRC01-class antibodies can bind to such trimers would further support the use of core-g28v2 60mer as a first boost and would identify potential second-boost candidates. We tested VRC01-class mAbs isolated after core-g28v2 60mer boosting (protein or mRNA) for binding to a panel of MD39-stabilized (17) HIV Env trimers that were highly sensitive to neutralization by VRC01-class bnAbs, according to available data on CATNAP (50–53). The N276 glycan was removed from these trimers by introducing N276D, N276Q, or T278M mutations. VRC01-class mAbs from both protein- and mRNA-immunized animals bound to N276-lacking trimers with similar affinities (Fig. 7A and B). Higher percent binders and higher affinity was noted for the N276D version of 191084 over the N276Q version and for the T278M version of HIV_001428_2 (hereafter referred to as 001428) over N276Q, indicating preferences for D276 and M278 (Fig. 7A and B). Both D276 and M278 are present in core-g28v2, and D276 is also present in eOD-GT8, likely contributing to the mutation preference. Fifteen of the VRC01-class mAbs elicited after boosting with core-g28v2 60mer mRNA were assessed for neutralization against a panel of N276-lacking and corresponding wildtype pseudoviruses (Fig. 7C). Neutralization was detected against N276D, T278M, and N276Q pseudoviruses, but not against wildtype pseudoviruses (Fig. 7C). Although neutralization activity was detected for mAbs, neutralization activity in polyclonal IgGs isolated from serum after boosting with core-g28v2 60mer was detected in only two of six animals tested (fig. S16). Thus, at least a subset of VRC01-class responses elicited by core-g28v2 60mer mRNA boosting showed detectable affinity for heterologous native-like trimers lacking the N276 glycan and had the capacity to neutralize corresponding pseudoviruses.

## DISCUSSION

Learning how to induce bnAbs against HIV by vaccination represents both a critical goal for global public health and a major challenge for immunology and vaccinology. Germline-targeting vaccine design provides a promising strategy for priming bnAb naïve precursors and shepherding them into acquiring the somatic mutations required for neutralization breadth and potency. The IAVI G001 clinical trial provided proof-of-principle that a germline-targeting priming immunogen could activate diverse bnAb naïve precursors (26). We used mAbs isolated from IAVI G001 to guide the selection of a first-boost immunogen, core-g28v2 60mer. We then showed, in a mouse model approximating human conditions of precursor frequency, affinity, and diversity, that VRC01-class B cells primed by eOD-GT8 60mer and boosted by core-g28v2 60mer acquired additional key VRC01-class residues and gained affinity for heterologous HIV Env trimers lacking the N276 glycan and also for a core-g28v2 variant containing the N276 glycan. The key VRC01-class residues within the HCDR2 induced by core-g28v2 60mer boosting are known to be important for neutralization breadth and potency (49). Furthermore, the correlations between affinity for core-g28v2 and both key mutations and SHM demonstrated that the structure of core-g28v2 selected for favorable directional maturation. Thus, we have demonstrated that a suitably designed boost immunogen that is closer in structure to the native glycoprotein than the prior immunogen and that possesses an affinity gradient in which bnAbs have higher affinity than precursors, can drive maturation toward bnAb development in an animal model. These findings provide important support for the germline-targeting vaccine strategy and represent important steps toward the goal of bnAb induction.

We note that core-g28v2 60mer was an effective boost after a single priming immunization by eOD-GT8 60mer in this mouse model even though our SPR analysis showed that only 56% or 28% of post-GT8 mAbs at week 6 (the timepoint for the core-g28v2 60mer boost) had detectable affinity for core-g28v2, for protein or mRNA priming, respectively, and among those binders, the geomean affinities for core-g28v2 were 12 μM and 8 μM, respectively. These data showed that successful boosting of bnAb precursors could be achieved even when only a subset of the previously primed precursors had measurable monovalent affinity for the booster immunogen and even if that affinity was relatively low. Whether this proves to be a general rule for booster design remains to be determined through additional studies, including for core-g28v2 60mer mRNA boosting in humans in IAVI G002, and also for other boost candidates for various bnAb classes in different animal models or humans.

There are several limitations to our study. First, none of the vaccine regimens elicited bnAbs that would be necessary for an effective vaccine. However, the goal of germline-targeting priming followed by sequential boosting is to initiate B cell responses that can mature into bnAbs and guide those B cells to acquire the appropriate mutations. Priming with eOD-GT8 60mer followed by boosting with core-g28v2 60mer successfully initiated VRC01-class B cell responses and guided the responses to acquire additional key mutations that allowed binding to more native-like antigens. Secondly, the VRC01-class precursor frequency in the SE09 mouse was 17 times higher than in humans; however, the VRC01-class precursors were still rare (1 in 13,600 naïve B cells), highly diverse with different CDR3s and light chains, and had affinities for eOD-GT8 that were comparable to human VRC01-class precursors. Boosting MBCs in mice to re-enter the germinal center is highly inefficient (54) and likely requires a higher precursor frequency present at priming compared to humans to generate sufficient MBCs as targets for boosting. Finally, the elevated frequencies of B cells with human V_H_1–2 and V_K_1–33 in the SE09 mouse reduced the genetic diversity of potential competitor B cells. However, we identified ample CD4bs epitope-specific, high-affinity, non-VRC01 antibodies among randomly sampled antigen-specific non-VRC01 antibodies elicited after eOD-GT8 60mer priming or core-g28v2 60mer boosting. This indicated that epitope-specific competition was present in the SE09 mouse model.

mRNA vaccine technology will likely prove essential for HIV vaccine development, as the favorable immunogenicity combined with increased speed and lower cost of producing clinical material should improve the feasibility and timelines of clinical trials testing multiple immunogens in series. For that reason, we compared adjuvanted protein immunization, as in IAVI G001, to mRNA immunization, and we found that mRNA performed at least as well, if not slightly better than, protein. This not only provided preclinical support for the IAVI G002 clinical trial evaluating eOD-GT8 60mer mRNA priming followed by core-g28v2 60mer mRNA boosting, but also demonstrated the feasibility of using mRNA to deliver self-assembling nanoparticle immunogens in vivo. Germline-targeting priming and sequential heterologous boosting with protein immunogens in highly permissive mouse models has been shown to induce increased SHM (21–23, 55) and to produce bnAbs (17, 18, 38). The critical differences in our study are that we have demonstrated maturation under more stringent, human-like conditions of precursor frequency, affinity, and diversity; we have validated mRNA as a viable delivery platform for germline-targeting priming and boosting; and our results build directly on the IAVI G001 trial leading directly to the IAVI G002 trial. Overall, our results predict favorable outcomes in IAVI G002 and suggest that the germline-targeting strategy combined with mRNA vaccination have promise for HIV vaccine development. These endeavors to induce VRC01-class bnAbs also provide guidance for application of the germline-targeting strategy to induce other bnAb classes to HIV and to other antigenically diverse pathogens.

## Materials and Methods

### Study Design

The main objectives of this study were to evaluate first-boost immunogens following eOD-GT8 60mer priming for their capacity to induce increases in VRC01-class B cell frequency, SHM, key mutations, and affinity and to compare adjuvanted protein versus mRNA immunization for delivery of self-assembling nanoparticle immunogens for prime and boost. The number of mice in each group was limited by mouse availability and the costs of analysis; however, the number of mice used was judged to be sufficient to detect clear differences between groups. Experiments were conducted once with group sizes ranging from 5 to 15 mice. Mice were randomly assigned to groups. Blinding was not used. Samples were excluded from analysis if fewer than 50,000 CD19^+^ B cells were detected during fluorescence activated cell sorting (FACS), indicating poor viability.

### Statistical analysis

Individual-level data for experiments where *n* < 20 are presented in data file S1. Except in figure S9, significant differences between groups were calculated with Kruskal-Wallis test followed by Dunn’s test for multiple comparisons with an alpha of 0.05 and shown as: ns, no significant difference, P>0.05; *P<0.05; **P<0.01; ***P<0.001; and ****P<0.0001. In figure S9, statistical comparisons were made by Mann-Whitney test and shown as *p<0.05, **p<0.01, ns, not significant. Correlation analysis was performed with simple linear regression of the log_10_ transform of the core-g28v2 K_D_ versus V_H_ %SHM, V_K_ %SHM, or key VRC01-class HC residues. All statistical analysis were performed using GraphPad Prism v9.5.1.

## Supplementary Material

Supplementary Materials

Data_S1

## Data Availability

All data associated with this study are in the paper or supplementary materials. BCR sequences from naïve and immunized SE09 mice and SPR K_D_ values for antibody-antigen interactions measured in this manuscript, are available in the public data repository https://github.com/SchiefLab/Cottrell2024 permanently archived at https://zenodo.org/records/10622207. Plasmids or proteins related to the immunogens, sort reagents, or antibodies employed in this study are available from WRS (schief@scripps.edu) under a material transfer agreement with The Scripps Research Institute. mRNA vaccine constructs can be made available from SH (Sunny.Himansu@modernatx.com) under a material transfer agreement with Moderna.
