# Supplementary material for: Heterologous prime-boost vaccination drives early maturation of HIV broadly neutralizing antibody precursors in humanized mice
Source: Sci Transl Med. Author manuscript; Available in PMC 2024 Jul 9. (PMC11233128; doi:10.1126/scitranslmed.adn0223)
Supplement: Supplementary Materials [file NIHMS2004404-supplement-Supplementary_Materials.docx]

**MATERIALS AND METHODS**

**Immunogen Design**

In this work we sought to develop boost immunogens to follow eOD-GT8 60mer priming. We began this booster design process before the IAVI G001 clinical trial had started, but we concluded our booster design process after we learned from our interim analyses of IAVI G001 that eOD-GT8 60mer could successfully prime VRC01-class responses in humans (*26*), and we were able to use post-GT8 mAbs from G001 to help select our boost candidate (Fig. 1). Here we describe the full arc of the design process.

We previously developed and tested first boost candidates to follow eOD-GT8 60mer priming using a relatively permissive knockin mouse model (VRC01gH, (*34*)). Boost candidates were BG505-GT3-core 60mer and BG505-GT3-SOSIP (*21*) and also HxB2 core-e-2cc N276D 60mer (*34*). However, we were concerned that the permissive nature of this knockin mouse model might be misleading (reduced competition due to about 85% usage of VRC01gH heavy chains, and potentially easier and more consistent SHM because all bnAb precursors had the same heavy chain that included the mature bnAb HCDR3); specifically we were concerned that boost candidates selected in the VRC01gH mouse might not perform well in more stringent mouse models or in humans.

Prior to having post-GT8 mAbs from G001, we tested the binding of our boost candidates to eOD-GT8-induced VRC01-class antibodies from the more stringent V_H_1-2 mouse model (*22*) and found no detectable binding (fig. S17A), suggesting that these boost candidates had little promise in the more stringent mouse or in humans. We found that eOD-GT6 (*14*) and the more engineered eOD-GT7 (*16*) both had affinity for post-GT8 VRC01-class Abs from the V_H_1-2 mouse (fig. S17A), suggesting that eOD-GT6 60mer or eOD-GT7 60mer might serve as a boost. However, we wanted to develop boost candidates that would be more different from the eOD-GT8 prime and at least somewhat more native-like in structure. We therefore initiated development of modified eODs and cores, aiming to identify a boost that succeeded in the VH1-2 mouse (or at least had affinity for post-GT8 VRC01-class Abs from that mouse), postulating that this would be our best candidate for human testing. We also developed stabilized, N276-lacking versions of native-like trimers from isolates highly sensitive to VRC01-class neutralization, such as the isolate 191084 (fig. S2, (*21*)), as even more native-like potential boost immunogens. We also carried out an eOD-GT8 60mer immunization study in Kymab mice as a follow-up to our original study (*42*); and this follow-up study further informed our booster design. Kymab mice have extremely low frequencies of VRC01-class precursors and therefore produce very low frequencies of VRC01-class responses to eOD-GT8 60mer (*42*), so we hypothesized that the Kymab model was not well-suited for evaluating boosters for VRC01-class responses; however, we also hypothesized that the Kymab model might give insights into how the human antibody repertoire might respond to eOD-GT8 60mer in terms of off-target non-VRC01-class responses and their cross-reactivity to boost candidates. In the follow-up study we delivered two shots of eOD-GT8 60mer six weeks apart and, two weeks after the second shot, we detected similar amounts of serum antibody binding to HxB2 core-e-2cc N276D and a VRC01 epitope-knockout version of HxB2 core-e-2cc N276D (KO11b), indicating that human immunoglobulin non-VRC01-class responses primed by eOD-GT8 were binding to HxB2 core-e-2cc N276D. We hypothesized that such non-VRC01-class serum Ab binding might impede boosting of VRC01-class responses in general, as we had previously noted (*21*); hence we aimed to minimize non-CD4bs cross-reactivity between our boost immunogens and eOD-GT8.

For new eOD-based boost candidates, we started from eOD-GT6 (*14*). The design goals were to make the CD4bs more native-like by removing selected germline-targeting mutations present in GT6 and to minimize reactivity to GT8-induced, non-VRC01-class, CD4bs-specific and non-CD4bs-specific Abs. Removing GT mutations was expected to reduce affinity for GT8-primed Abs, so we took a stepwise approach to identify constructs that retained as few GT mutations as possible while also retaining at least low detectable affinity for GT8-primed Abs from the VH1-2 mouse. This resulted in a series of eOD-GT6 variants termed eOD-GT6v2, eOD-GT6v3, and eOD-GT6v4 (*26*). These GT6 variants eliminated 2, 3, and 4 GT mutations, respectively (see fig. S38 in Leggat *et al*. (*26*)). We hypothesized that these changes to the VRC01 epitope would reduce reactivity to GT8-induced, non-VRC01-class, CD4bs-specific Abs. We focused our efforts on eOD-GT6v2, as that molecule generally had better affinities for GT8-induced VRC01-class Abs from the VH1-2 mouse. To reduce reactivity to GT8-induced non-CD4bs Abs, we employed Rosetta to computationally resurface eOD-GT6 outside the CD4bs and outside the existing glycosylation sites. This resulted in resurfaced variants of the above-mentioned GT6 variants, including resurfaced eOD-GT6v2, termed eOD-GT6v2-cRSF.

For new core-based boost candidates, we started from HxB2 core-e-2cc N276D (*34*). The design goals were (i) to maintain or improve affinity for eOD-GT8 60mer-induced VRC01-class Abs from the VH1-2 mouse model; (ii) to maintain a strong affinity gradient in which mature VRC01-class bnAbs bind more strongly than post-GT8 VRC01-class mAbs; (iii) to minimize boosting of GT8-induced, non-VRC01-class, CD4bs-specific and non-CD4bs-specific Abs (iv); to minimize priming of non-CD4bs (off-target) responses; (v) to ensure robust shared CD4 T help with eOD-GT8 60mer, by utilizing the same lumazine synthase nanoparticle as employed for eOD-GT8 60mer; (vi) to ensure good thermal stability and nanoparticle formation; and (vii) to increase the content of shared sequence with HIV Envelope (Env) trimers so as to provide potential T-helper priming prior to a subsequent trimer boost immunization. To achieve these goals, we aimed to develop a core with the CD4bs as native-like as possible that would still allow binding to GT8-induced VRC01-class Abs from the VH1-2 mouse; with the non-CD4bs surface differing as much as possible from eOD-GT8 (by resurfacing) and as non-immunogenic as possible (by V3-loop minimization and as much glycan-masking as possible); and with good thermal stability and nanoparticle expression. Starting from HxB2 core-e-2cc N276D, we carried out multiple iterations of V3-loop truncation (to minimize potential immunogenicity of the already reduced V3 loop present on HxB2 core-e-2cc N276D), computational resurfacing (to reduce antigenic similarity to eOD-GT8 outside the CD4bs), and glycan-masking (to minimize immunogenicity outside the CD4bs). Analogous modifications were made in the context of an extended version of HxB2 core-e-2cc N276D (TH6 version), in which the N- and C-termini were extended and two internal segments were adjusted to increase the content of sequence shared with largely conserved, non-glycosylated regions of HIV Env trimers, as an attempt to provide for potential shared T-help between our core booster and any subsequent trimer boost. Modified versions of HxB2 core-e-2cc N276D led to core-g5, and modified versions including the TH6 extensions and internal changes led to core-g28 and core-g28v2.

Modifications were generally designed using Rosetta, starting from an initial homology model of HxB2 core-e-2cc N276D based on the structure of mature VRC01 bound to a core-e gp120 (PDB: 3ngb). The different types of modifications were tested alone and in combinations. In the early iterations, only monomers were produced and tested, but in the later stages both monomers and 60mers were produced and tested. Readouts included: (i) fundamental biophysical readouts of expression amount, solution multimeric state by SECMALS, and thermal stability; (ii) surface plasmon resonance (SPR) affinities for mature VRC01-class antibodies; and (iii) SPR affinities for GT8-induced VRC01-class antibodies from the VH1-2 mouse (antibodies were from Tian *et al*. 2016 (*22*) and from a 2018 in-house experiment in which a single immunization of eOD-GT8 60mer was delivered with the Sigma adjuvant system and day 35 GT8^++^/KO^-^/IgG^+^ memory and germinal center B cells were sorted into 96-well plates and their BCR sequences were obtained by reverse transcription polymerase chain reaction and Sanger sequencing.) We also tested our late-stage core boost candidates for binding to GT8-induced VRC01-class and non-VRC01-class antibodies from G001 once those antibodies became available starting in 2020.

For V3-loop truncation, we employed Rosetta KIC (*57*) to model loops of lengths 2 to 6 aa, with the aim of introducing a glycosylation site within the V3. One thousand models were generated for each loop length. The final candidates, selected based on loop closure score and total energy, were produced in vitro and evaluated for expression amount, stability and binding to a panel of VRC01-class bnAbs (VRC01, 12a21, 12a12, CHA31, PGV04, 3BNC60, 3BNC117, VRC07, PG19, PGV20) and the non-nAb B6. In the final selected V3-loop minimization design, the starting v3 loop sequence “RPNNGGSGSGGNMRQ” was substituted with a 6-residue loop sequence “AGNGTA”, which incorporated an engineered glycosylation site, referred to as position “300*”, with the star signifying that this position is present only on an artificial truncated loop. This modified V3-loop was incorporated into the final core-g5, core-g28, and core-g28v2 designs.

For glycan masking, we started by including two glycans (N206 and N246) from a previously reported hyperglycosylated core called 6G (*58*). To identify additional sites for glycosylation, we focused on surface residues located at least 5 Å away from the CD4bs (HxB2 numbering 92-97, 276-282 and 455-476) and at least 5 Å away from the asparagine residues within existing glycosylation sites. Using Rosetta, we evaluated the energy difference resulting from the introduction of the Nx(S/T) sequon, where x is any amino acid except proline. Mutations that did not destabilize the protein were deemed suitable for experimental evaluation. For the initial design round, we incorporated only single glycan mutations. Each glycan variant was screened for expression and antigenicity against a panel of mature VRC01 antibodies. Promising glycan candidates were chosen and combined for the subsequent rounds of optimization, and glycosylation designs were also combined with V3-loop minimization and resurfacing designs as those modifications were being developed iteratively. In the design of glycosylation sites, we also attempted to utilize NxT sequons whenever possible, as they have been shown to be glycosylated more efficiently than NxS sequons (*59-61*). In core-g5, which includes the final V3-loop minimization design and resurfacing, there are 8 engineered glycosylation sites (at positions 63, 82, 113, 202, 206, 246, 300, and 441 in HxB2 numbering) and 16 native glycosylation sites (at positions 88, 230, 234, 241, 262, 289, 295, 332, 339, 356, 386, 392, 397, 406, 448 and 463 in HxB2 numbering). In core-g28v2, which includes the final V3-loop minimization design and resurfacing in the context of the TH6 extended core to increase potential shared T-helper epitopes with HIV Env trimers, there are a total of 28 glycosylation sites, with 12 engineered sites (at positions 113, 120, 300*, 344, 402, 409, 413, 419, 423, 434, 439, and 442 in HxB2 numbering) and 16 native sites (at positions 88, 230, 234, 241, 262, 289, 295, 332, 339, 356, 386, 392, 397, 406, 448, and 463 in HxB2 numbering). Core-g28 was the same as core-g28v2, except that core-g28 contained an engineered glycosylation site at 399 instead of the native glycosylation site at 397. We found that core-g28v2 60mer demonstrated superior nanoparticle formation (fig. S3), with a higher fraction of nanoparticles and greater homogeneity, compared to core-g28 60mer, which was the primary reason that we selected core-g28v2 over core-g28 for clinical testing. Subsequently, we also found that core-g28v2 monomer exhibited superior binding to GT8-elicited antibodies from the G001 study.

For core resurfacing, we identified surface positions with identical or similar amino acids in both eOD-GT8 and HxB2 core-e-2cc N276D (419, 462, 264, 267, 268, 270, 275, 278, 279, 298, 334, 335, 340, 344, 347, 351, 360, 362, 363, 381, 382, 444, and 457), and we used RosettaScripts (*62*) to design new amino acids at those positions. Minimal backbone movement was allowed. To guide the sequence design process, a position-specific scoring matrix (PSSM) was generated by aligning HIV Env trimer sequences from the LANL database (www.hiv.lanl.gov); only amino acids present in native Env trimers were permitted, and an energy bonus was given to the native amino acid to reduce the likelihood of introducing destabilizing mutations. Most resurfaced designs were tested as fully resurfaced proteins, but for selected positions within the edge or near the CD4bs, individual point mutations were tested. Resurfacing modifications were found to affect thermal stability and nanoparticle formation propensity, with some designs reducing stability by as much as 10° and correspondingly lacking the ability to form nanoparticles, and other designs improving both thermal stability and nanoparticle formation. Although this resurfacing effort was almost entirely focused on making mutations at non-CD4bs positions, we did allow Rosetta to make substitutions at other positions, including position 278 within loop D at the edge of the CD4bs, because an additional study of non-VRC01-class responses to eOD-GT8 60mer in Kymab mice (*63*) had identified loop D as an important target of CD4bs-specific non-VRC01-class responses. Computational resurfacing identified the mutation R278M as the most favorable mutation at that position. This mutation had previously and independently been selected by a yeast display library screening method of developing a resurfaced eOD-GT8 (*63*); and we found that the T278M mutation was effective at reducing binding to GT8 of CD4bs-specific non-VRC01-class antibodies induced by GT8 in Kymab mice (*63*), hence we hypothesized that including T278M in our core booster might reduce binding of such antibodies to core. Although we found experimentally that the T278M mutation was slightly destabilizing on core proteins (reducing thermal stability by about 1°C), we also found that the T278M mutation improved binding to mature VRC01-class bnAbs and to GT8-induced VRC01-class antibodies from IAVI G001 (fig. S17B). Given the beneficial VRC01-class antigenicity and the potential for reducing boosting of CD4bs-specific non-VRC01-class responses primed by GT8, this mutation was retained in our final designs. The core-g5 design included a total of 13 resurfacing mutations, and the core-g28 and core-g28v2 designs each included a total of 8 resurfacing mutations.

In this work we also sought to make in vivo comparisons between core-g28v2 and c13.G4.2 (*38*), which had previously been reported as a ferritin nanoparticle-based immunogen. To enable direct comparisons, we produced c13.G4.2 60mers using the same lumazine synthase platform we have used for core-g28v2, and we also produced core-g28v2 ferritin nanoparticles, using the same ferritin platform previously described for c13.G4.2 (*38*).

**Lumazine synthase stabilization**

All lumazine synthase nanoparticles tested in this study utilized the previously reported d41m3 version of lumazine synthase. This version includes two engineered disulfides and three mutations to disable the enzymatic active site (*42*). When eOD-GT8 60mer was evaluated previously for delivery by an RNA replicon platform, the d41m3 version was found to perform better than the original version (*64*).

**VRC01-class epitope knockout mutants**

The eOD-GT8 KO used here was eOD-GT8 KO11 (with mutations 280R, 365L, and 371R) (*65*). The core-g28v2 KO used here was core-g28v2 KO11b. This version has the same mutations as KO11 but also included D368R.

**Molecular modeling**

A de novo model for core-g28v2 was generated using AlphaFold2. Man9 glycans were added and relaxed using Rosetta (*66-68*). Figures were made using UCSF Chimera (*69*).

**Protein production**

His-tagged and His-Avi-tagged monomeric and trimeric antigens were produced by transient transfection of HEK-293F cells (Thermo Fisher) and purified by immobilized metal ion affinity chromatography (IMAC) using HisTrap excel columns (Cytiva) followed be size-exclusion chromatography (SEC) using either Superdex 75 10/300 GL or Superdex 200 Increase 10/300 GL columns (Cytiva). The molecular weight and the homogeneity of antigens were confirmed by size-exclusion chromatography-multi-angle light scattering (SEC-MALS) in phosphate-buffered saline (PBS) using Superdex 75 10/300 GL or Superdex 200 Increase 10/300 GL columns (Cytiva) columns operating with an isocratic flow of 0.5 mL/minute followed by DAWN HELEOS II and Optilab T-rEX detectors (Wyatt Technology). His-Avi-tagged antigens were biotinylated using BirA (Avidity) and purified again to remove excess biotin using SEC with either Superdex 75 10/300 GL or Superdex 200 Increase 10/300 GL columns (Cytiva).

Nanoparticle 60mer immunogens were produced by transient transfection of HEK-293F cells (Thermo Fisher). Immunogens were then purified by *Galanthus nivalis* lectin affinity chromatography (Vectorlabs) followed by SEC using a Superose 6 16/600 PG column (Cytiva). Immunogen preps confirmed to contain < 5 EU/mg of endotoxin using an Endosafe instrument (Charles River).

Genes encoding the antibody variable fragment (Fv) regions were synthesized by GenScript and cloned into antibody expression vectors pCW-CHIg-hG1 and pCW-CLIg-hk. Monoclonal antibodies were produced using transient transfection of HEK-293F cells (Thermo Fisher). They were then purified using rProtein A Sepharose Fast Flow resin (Cytiva).

**Site specific glycan profiling of core-g28v2 60mer**

Engineered N-linked glycosylation sites are frequently under-occupied. To assess the glycosylation profile of the core-g28v2 60mer immunogen, site specific glycan profiling was conducted as previously described (*70*). The degree of glycan occupancy and proportion of glycans that were complex and oligomannose/hybrid type were determined.

**Immunizations in SE09 Mice**

All work followed IACUC guidelines associated with animal protocol number 20-0001. Mice were injected with 10µg (50µl total volume) of mRNA intramuscularly (I.M.) under anesthesia (5% isoflurane induction) in the left quadriceps muscle. All primes and subsequent boosts were done in the same location. Protein injections (20µg, 200µl total volume) were performed intraperitoneally (I.P.), utilizing 100 µL of the Sigma Adjuvant System (Cat#S6322). I.P. injections consisted of two 100 µL injections given on either side of the abdomen. Insulin syringes were used for all injections (BD #328440).

**Tissue harvest and serum isolation**

Mice were euthanized with compressed CO_2_ (100%) in a clear chamber to allow for visualization of respiration and subsequent death through respiratory cessation. Blood was collected from the chest cavity prior to the removal of the spleen and lymph nodes (mesenteric, inguinal, and popliteal (RNA injections only, left leg only). Tissues were placed in 3 mL resuspension buffer (1x PBS Ca/Mg^++^ free, 1mM EDTA, 25mM HEPES, pH 7.0, 1% heat-inactivated fetal bovine serum [FBS]) in a 15 mL polypropylene tube on ice. Tissues were disassociated using the rough ends of two sandblasted microscope slides in a 5mL petri dish, then returned to the same 15 mL polypropylene tube for centrifugation (460xg for 5 minutes at 4°C). Red blood cell lysis was performed using 1 mL of ACK buffer (Quality Biological, Cat#118-156-721) for 2 minutes on ice in a 15 mL polypropylene tube. Lysis was halted by adding 14 mL resuspension buffer per sample. Post lysis and centrifugation (460xg for 5 minutes), cells were resuspended in 3 mL Bambanker freezing medium (Bulldog Bio, Cat# BB01) prior to filtration through a cotton-plugged, borosilicate Pasteur pipette into a borosilicate glass test tube. 1 mL filtered-cell solution was subsequently divided into three cryovials per mouse, which were precooled in a Styrofoam rack on dry ice. Cells were stored at -80°C for 2 to 7 days prior to long-term storage in liquid nitrogen. Serum samples were collected by spinning the blood at 14,000 RPM for 30 minutes. Serum samples were stored at -20°C.

**Sample preparation and B cell sorting**

For immunized samples, frozen splenocytes and lymphocytes were thawed in 10 mL 50:50 heat inactivated FBS (Omega Scientific, cat# FB-02):RPMI-1640 (Gibco, cat# 61870-036) pre-warmed to 37°C. Unimmunized splenocytes for naïve B cell sorting were used fresh after processing. Cells were centrifuged at 400xg for 5 minutes. After the supernatant was removed, cells were resuspended in 3 mL FACS buffer (1% v/v heat inactivated FBS, 1 mM EDTA (Invitrogen, cat# 15575-038), 1 mM HEPES (Gibco, cat# 15630-080) in Dulbecco’s PBS (DPBS; Corning, cat # 21-031-CV)), and enumerated. Because cells in SE09 mice constitutively express green fluorescent protein (GFP), in some cases a small amount of cells were set aside for GFP compensation. After counting, cells were subjected to B cell isolation using the StemCell) EasySep Mouse Pan-B Cell Isolation Kit (StemCell), cat #19844A) according to manufacturer-provided instructions.

Streptavidin (SA) conjugated-baits were prepared by combining biotinylated monomeric baits or Env trimer baits with fluorescent SA at room temperature for at least 1 hour in the dark. Wild-type baits were complexed with SA-Alexa Fluor 647 (Invitrogen, cat# S21374) and SA-brilliant violet (BV) 421 (BioLegend, cat# 405225). Knockout (KO) baits, if used, were conjugated with SA-phycoerythrin (PE)-cyanine (Cy) 7 (BioLegend, cat# 405206). Monomeric baits were conjugated with SA at a 4:1 (bait:SA) ratio and used at a final bait concentration of 200 nM for staining. Env trimer baits were conjugated with SA at a 2:1 (bait:SA) ratio and used at 100 nM.

Isolated B cells were transferred over to 15 mL conical tubes, washed once with FACS buffer, and stained with 100 µL antibody cocktail mix consisting of PE anti-CD19 (BD Biosciences, cat# 553786), BV786 anti-IgM (BD Biosciences, cat#743328), Peridinin chlorophyll protein (PerCP)-Cy5.5 anti-IgD (BD Biosciences, cat# 564273), allophycocyanin (APC)-Cy7 anti-F4/80 (BioLegend, cat # 123118), APC-Cy7 anti-CD11c (BD Biosciences, cat# 561241), APC-Cy7 anti-Ly-6C (BD Biosciences, cat# 557661), APC-H7 anti-CD8a (BD Biosciences, cat# 560182), and APC-H7 anti-CD4 (BD Biosciences, cat# 560181). All antibodies were used at 1:100 dilution. When only wild type (WT) baits were used, the prepared fluorescent SA-baits were added along with the antibody master mix for 30 minutes at 4°C in the dark. When using KO baits, fluorescent SA-KO baits were first added to cells with the antibody master mix for 15 minutes, followed by the addition of WT baits for an extra 30 minutes. During the addition of antibody master mix, a unique TotalSeq-C anti-mouse hashtag antibody (BioLegend) was added to each sample at a concentration of 2.5 μL / up to 20 million cells. Following antibody staining, 1:300 1 mL live/dead stain (LIVE/DEAD Fixable Aqua, Invitrogen, cat# L34966) was added to each sample and incubated for an additional 15 minutes at 4°C. At the end of staining, cells were washed with 10 mL of FACS buffer, and resuspended in 500 μL FACS buffer.

All samples were sorted on a BD FACSMelody. Single color compensations were performed using C57BL/6 splenocytes with matched antibodies. For channels used for bait detection, cells were stained with biotinylated anti-CD19 (BioLegend, cat# 115503) followed by secondary staining with the appropriate fluorescent SA. Samples were filtered through a 35 μm mesh-cap FACS tube (Falcon, cat# 352235) prior to being loaded on the sorter. A maximum of 15,000 cells were sorted using purity mode into a PCR plate well containing 20 μL of 0.2 μm filtered FBS. Event rates were typically maintained at about 1000 events per second and no more than 1500 events per second to ensure high sorting efficiencies.

**BCR sequencing using 10x Genomics**

Sorted samples were prepared for BCR sequencing by the 10X Genomics Single Cell Immune Profiling platform. After cell sorting, DPBS was added up to near top of the sample collection well (about 100 μL) and gently mixed to dilute the FBS catch buffer. The plate was sealed and cells were centrifuged for 2 minutes at 2000 rpm, after which the excess buffer was removed except for about 38 μL required for the 10X Genomics GEM reaction. Samples were processed according to manufacturer’s user guide for Chromium Next GEM Single Cell 5’ Reagent Kits v2 (Dual Index) with Feature Barcoding, with two main modifications. The number of PCR cycles in the cDNA amplification step were determined by assuming that only 20% of the total number of cells sorted would be recovered. This modification was made based on the observation that on average, the number of unique paired-BCR sequences recovered from the 10X Genomics platform was typically about 20% of the total number of cells sorted. In the second modification, the number of PCR cycles for each of the V(D)J amplification steps were increased to 10 cycles if the number of cells sorted (according to the sorter) was fewer than 1000 cells. Details regarding the modified protocol can also be found in Hurtado *et al.* (*71*). Pooled libraries were sequenced on an Illumina NextSeq 2000 using a 100-cycle P3 reagent kit (Illumina, cat# 20040559) with a target depth of 5000 paired-end reads for both the V(D)J and Feature Barcode Libraries and run using read parameters indicated in the 10x Genomics user guide.

**Sequence analysis**

Raw sequencing data were demultiplexed, processed into assembled VDJ contigs and counts matrix files, and assigned to specific animal IDs based on TotalSeq-C antibody hashtag counts using Cell Ranger (v6.1) and scab as previously described (*71*). Gene assignment, annotation, and formatting into Adaptive Immune Receptor Repertoire (AIRR) format (*72*) for paired heavy and light chain antibody sequences was performed using Sequencing Analysis and Data library for Immunoinformatics Exploration (SADIE) with a custom SE09 mouse germline reference database (*26*). VRC01-class identification and mutational analysis was performed using the SADIE renumbering module by numbering each sequence using Kabat numbering and checking for the following key VRC01-class residues: (i) germline: 47W, 50W, 55G, 71R; (ii) non-paratope: K19R, G31D/A, Y33I/V/T, M34I/L, S76E/D, S82aK/R; and (iii) paratope: N52K/R, N53/R/Q/K/L/M/V/E, S54Y/G/H/F/R, G56A, T57V, Q61R/H/G, K62Q/G, T73V/I, S74Y, Trp_103-5_.

The frequency of VRC01-class memory B cells (MBCs) was calculated by multiplying the frequency of antigen-specific MBCs among all MBCs processed by FACS and the frequency of VRC01-class BCRs among all sequenced heavy/light pairs. Similarly, the frequency of VRC01-class with human V_K_1-33 light chains was calculated by multiplying the frequency of antigen-specific MBCs among all MBCs processed by FACS and the frequency of VRC01-class^VK1-33^ BCRs among all sequenced heavy/light pairs.

**ELISA**

ELISA plates (Corning 96-Well Half-Area Plates, Catalog # 3690) were directly coated with ELISA antigens at 2 µg/mL on Day 1. Plates were incubated overnight at 4°C. Plates were washed three times with PBST (PBS + 0.2% tween 20) and blocked with PBST containing 5% skim milk (BD Difco Skim Milk Catalog # 232100) and 1% FBS (Thermo Fisher, Catalog # 16000044) for 1 hour at room temperature on Day 2. Plates were then washed three times and 25μL of serum serially diluted (1:1000) in blocking buffer (PBST, 1% [w/v] FBS) was added for 1 hour at 37°C and 80% humidity. Plates were washed five times and 25 μL of Anti-Mouse IgG (H+L) (Jackson ImmunoResearch Catalog # 115-035 166) was added at 1:5,000 dilution in PBST + 1% FBS. After a 1 hour incubation at room temperature, plates were washed three times and TMB Chromogen Solution (Thermo Fisher Catalog # 002023) substrate was added. To stop the reaction, 25 μl 0.5 M H2SO4 were added after 5 minutes. Absorption was read at 450 and 570 nm on a Molecular devices VersaMax plate reader (VersaMax). Background subtraction was performed by subtracting the 570 nm value from the corresponding 450 nm value. Data were subsequently analyzed in GraphPad Prism v9.5.1. using the Agonist vs response – variable slope equation to calculate the 50% effective concentrations (EC_50_).

**SPR**

We measured kinetics and affinity of antibody-antigen interactions on Carterra LSA using HC30M or CMDP Sensor Chip (Carterra) and 1x HBS-EP+ pH 7.4 running buffer (20x stock from Teknova, Cat. No H8022) supplemented with bovine serum albumin (BSA) at 1 mg/mL. We followed Carterra software instructions to prepare the chip surface for ligand capture. In a typical experiment, about 2500 to 2700 resonance units (RU) of capture antibody (SouthernBiotech Cat no 2047-01) in 10 mM Sodium Acetate pH 4.5 was amine coupled. Phosphoric Acid 1.7% was our regeneration solution with 60 seconds contact time and injected three times per each cycle. The ligand solution concentrations were 1 to 5 µg/mL for the standard method used to assess monovalent analytes and 0.1 to 0.2 µg/mL for the low-capture IgG method used to assess binding to trimeric analytes. In both methods, the contact time was 3 to 5 minutes. Raw sensograms were analyzed using Kinetics software (Carterra), interspot and blank double referencing, Langmuir model. Analyte concentrations were quantified on NanoDrop 2000c Spectrophotometer using Absorption signal at 280 nm. Analyte samples were buffer-exchanged into the running buffer using dialysis. We typically covered a broad range of affinities in our runs and the best referencing practices are different depending on how fast the off-rate is for particular ligand. For fast off-rate (faster than 1e-2 1/s) we use automated batch referencing that includes overlay y-aline and higher analyte concentrations. For slow off-rates (9e-3 1/s or less) we use manual process referencing that includes serial y-align and lower analyte concentrations. After automated data analysis by Kinetics software, we also did additional filtering to remove datasets with highest response signals smaller than signals from negative controls. This additional filtering was performed automatically using a R-script.

**Serum IgG Isolation**

Polyclonal IgG was isolated from mouse serum samples using Protein G Sepharose 4 Fast Flow resin (Cytiva, 17-0618-05). Resin was washed 3 times in 1x PBS and resuspended at 1:1 ratio resin to PBS. 50 μL of resin/PBS mixture was added to 200 μL heat-inactivated mouse serum and incubated with agitation overnight at room temperature. Samples were loaded into empty spin columns (Pierce, 89868) and centrifuged at 5000xg for 2 minutes. Resin was washed 3 times with 1x PBS. IgGs were eluted into 50 μL 1M Tris pH 8 using 50 μL 0.1M glycine pH 2.7. IgGs were concentrated and buffer exchanged into 1X TBS using 30 kDa Amicon Ultra 0.5 mL centrifugal filters (Millipore, UFC503096).

**Competition biolayer interferometry (BLI)**

A competition BLI experiment was performed to assess epitope specificity of elicited VRC01-class mAbs. Biotinylated core-g28v2 monomer was loaded onto streptavidin OCTET biosensors (Sartorius, 18-5019) at 5 μg/mL in kinetics buffer (1xPBS, pH 7.4, 0.01% [w/v] BSA, and 0.002% [v/v] Tween 20) until a response of 1 nanometer shift was reached. Loaded biosensors were dipped into kinetics buffer for 1 minute to acquire a baseline and then moved to wells containing VRC01 Fab at 2 μM in kinetics buffer. The VRC01 Fab was allowed to saturate the core-g28v2 load biosensors for 2 minutes. The biosensors were then moved to wells containing competitor IgGs at 500 nM in kinetics buffer for 2 minutes. Control binding experiments were conducted in which no VRC01 Fab was used. Competition was apparent by a lack of signal for IgG binding when VRC01 Fab was used compared to the signal acquired when no VRC01 Fab was used. All BLI experiments were conducted at 25°C.

**Neutralization**

Pseudovirus neutralization assays were performed as previously described (*73*) with minor modifications. Briefly, single cycle infectious pseudoviruses were generated by co-transfecting HEK293T cells with Envs of interest with an Env deficient HIV-1 backbone plasmid pSG3ΔEnv using Fugene 6 (Promega, E2692), or PEI MAX (Polysciences, Inc, 24765-1). Viruses were harvested 72 hours post-transfection and kept frozen at -80°C until use. 50 μL of target TZM-bl cells at 100,000 cells/mL were distributed into half area 96 well plates (Corning, 3688) the day before the assay. On the day of the neutralization assay, mAbs were serially diluted in D10 media (Dulbecco's Modified Eagle Medium (Gibco, 10313-021), 10% FBS (Omega Scientific, FB-02), 1x PenStrep (Gibco, 15070-063), and 1x GlutaMAX (Gibco, 35050-061)). Viruses where prepared by thawing at 37°C, and concentrated or diluted with D10 according to previously defined titers. DEAE-dextran (Spectrum Chemical Mfg. Corp., DE132) was added to the viruses at 10 μg/mL. The prepared mAb dilutions and viruses were distributed into 96 well round bottom plates (Corning, 3788) at a 1:1 v/v ratio and incubated at 37°C for 1 hour. Following the 1 hour incubation, the supernatant was carefully aspirated from the prepared TZM-bl plates, then 25 μL of appropriate mAb:virus mixtures were added to the target cells and returned to the incubator. 24 hours later, 75 μL of D10 were added to each well and further incubated for an additional 48 hours. The supernatant was removed and TZM-bl cells were lysed in 45 μL/well of 1X Cell Lysis Buffer (Promega, cat# E4550) for 15 minutes, after which 30 μL/well of substrate was added (Promega, cat# E4550) and luminescence was read on a BioTek Synergy H1 Plate Reader. Half-maximal inhibitory concentration (IC_50_) values were interpolated from the One site Fit logIC50 model in Prism 9 (GraphPad). All curve fits were constrained between 0 to 100% neutralization. Both TZM-bl cells and HEK293T cells were cultured and maintained in D10 media in a 37°C humidity and CO_2_ controlled incubator.

**Fig. S1. Characterization of the V_H_1-2^JH2^/V_K_1-33^hTdT^ (SE09) mouse model. (A)** Illustration of genetic modifications in the *Igh* and *Igk* locus of the V_H_1-2^JH2^/V_K_1-33^h^*^TdT^* (SE09) rearranging mouse model. The mouse V_H_81X was replaced with the human V_H_1-2 and the mouse J_H_s were replaced with the human J_H_2. The intergenic control region 1 (IGCR1) regulatory element in the V_H_-to-D intervening region was deleted. The mouse V_K_3-2 was replaced with human V_K_1-33 plus a CTCF-binding element (CBE) 50 bp downstream of its recombination signal sequence. The human *TdT* gene was knocked into mouse *Rosa* locus. **(B)** Frequency of VRC01-class naïve B cells in humans (*16, 44, 45*) and in SE09 mice after sorting with eOD-GT8. Solid red lines indicate overall frequency (total VRC01-class naïve B cells among all samples divided by total number of naïve B cells sorted among all samples). **(C)** Frequency of VRC01-class naïve B cells with human V_K_1-33 LCs in humans (*16, 44, 45*) and in SE09 mice after sorting with eOD-GT8. Solid red lines in (B and C) indicate overall frequency. Dashed blue lines in (B and C) indicated median frequencies. **(D)** LCDR3 amino acid logo plots for VRC01-class BCRs. **(E)** Monovalent K_D_ values measured by SPR of VRC01-class naïve precursors isolated from humans and SE09 mice for binding to eOD-GT8. **(F)** Monovalent K_D_ values measured by SPR of VRC01-class naïve precursors with human V_K_1-33 light chains isolated from humans and SE09 mice for binding to eOD-GT8. Red lines in (E and F) denotes the median.

**
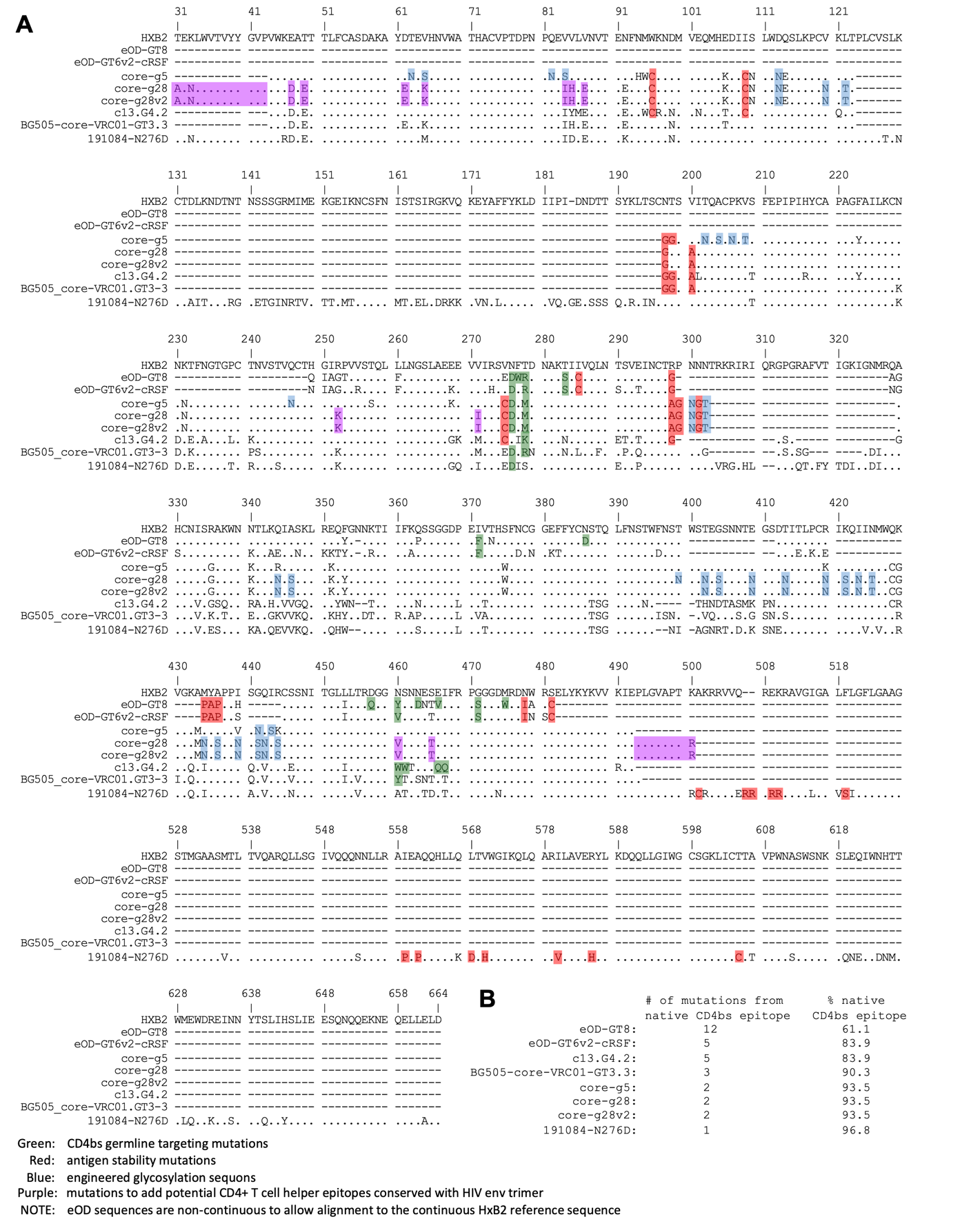
**

**Fig. S2. Sequence alignment of immunogens. (A)** Immunogens aligned to HxB2 reference sequence with CD4bs (green), stabilization (red), glycan masking (blue), and TH6 (purple) mutations highlighted. **(B)** Table showing the number of mutations within the CD4bs epitope and the corresponding percent native.

Fig. S3. Biophysical characterization of nanoparticle immunogens. (A) Normalized SEC profiles for core-g5 60mer, core-g28 60mer, and core-g28v2 60mer plotting normalized milli-absorbance unit (mAU) versus elution volume. (B) Computational model of core-g28v2 60mer showing lumazine synthase (pink), N-linked glycans (blue), CD4bs (yellow), and gp120 peptide (green). (C) Negative stain electron micrograph of purified core-g28v2 60mer. (D) Differential scanning calorimetry for assessing stability of core-g5 60mer, core-g28 60mer, and core-g28v2 60mer (red: raw data; blue: 2-state model fit). (E) Nanoparticle yield data following *Galanthus nivalis* lectin (GNL) affinity chromatography and SEC. (F) SPR data for non-VRC01-class mAbs from IAVI G001 samples. (G) SPR affinity data for core-g28v2, showing affinity gradient of human VRC01-class naïve precursors, eOD-GT8 induced VRC01-class mAbs (IAVI G001), and VRC01-class bnAbs. (H) SPR affinity data for VRC01-class naïve precursors with human V_K_1-33 light chains isolated from SE09 mice binding to eOD-GT8 and core-g28v2. (I) Glycan occupancy data for purified core-g28v2 60mer as determined by mass spectrometry. Red lines indicate medians in (F to H).

**Fig. S4. SPR affinity measurements for VRC01-class mAbs elicited after priming with eOD-GT8 60mer protein in SE09 mice and humans. (A)** K_D_ values were measured by SPR for mAbs elicited by the indicated first boost candidates. Data from Fig. 1C are included here to facilitate easy comparison among all booster immunogen candidates. Thick lines indicate median values, boxes show 25 and 75% quantiles. ^*^Low-capture IgG SPR method many include some avidity for trimeric analytes. **(B)** Table listing immunogens with valency and CD4bs epitope mutations.

**Fig. S5. Representative FACS gating scheme for the isolation of antigen specific MBCs and BCR sequencing workflow.**

**Fig. S6. Comparison of additional protein booster immunogens. (A)** Shown is the immunization scheme for evaluating additional boost immunogen candidates delivered as adjuvanted proteins in SE09 mice. **(B)** The frequency of antigen^++^ MBCs among total MBCs was analyzed by flow cytometry. Each group was sorted with matched antigens. **(C** to **E)** Shown is the frequency of VRC01-class MBCs among antigen^++^ MBCs (C), the frequency of VRC01-class MBCs among total MBCs (D), and the frequency of VRC01-class MBCs with human V_K_1-33 light chains among total MBCs (E). **(F** and **G)** The median percent amino acid SHM in the V_H_ gene (F) and in the V_K_/V_L_ genes (G) is shown for all VRC01-class MBCs. **(H and I)** The median percent amino acid SHM in the V_H_ gene (H) and in the V_K_/V_L_ genes (I) for all non-VRC01-class MBCs. Each point represents the median per mouse and the red bars indicate the median of medians for panels (B to I). The core-g28v2 60mer protein data from Fig. 2 is included in panels B through I to facilitate comparison. Statistical comparisons were made by Kruskal-Wallis test followed by Dunn’s test for multiple comparisons. *p<0.05, **p<0.01, ***p<0.001, ****p<0.0001; ns, not significant.

**Fig. S7. Key VRC01-class heavy chain residues in VRC01-class bnAbs and elicited after eOD-GT8 60mer protein immunization. (A)** Key VRC01-class heavy chain residues among VRC01-class bnAbs. **(B)** Key VRC01-class heavy chain residues among the GT8 protein primed group. Colors along the y-axis represent individual mice.

**Fig. S8. SHM analysis of VRC01-class BCRs induced by mRNA/LNP immunization. (A)** Median percent amino acid SHM in the V_H_ gene among VRC01-class MBCs induced after mRNA/LNP immunization. **(B)** Median percent amino acid SHM in the V_K_/V_L_ genes among VRC01-class MBCs induced after mRNA/LNP immunization.

**Fig. S9. Comparison of protein plus adjuvant immunization versus immunization with mRNA/LNPs.** Select data repeated from Fig. 2D, 2E, and 2F; Fig. 4C; Fig. 5C and 5D; Fig. 6A; and fig. S8A. **(A)** The frequency of VRC01-class MBCs among total MBCs after priming with eOD-GT8 60mer in a protein or mRNA/LNP format. **(B)** The frequency of VRC01-class MBCs with human V_K_1-33 light chains among total MBCs after priming with eOD-GT8 60mer. **(C)** Median percent amino acid SHM in the V_H_ gene among all VRC01-class MBCs after priming with eOD-GT8 60mer. **(D)** 90^th^ percentile key VRC01-class heavy chain residues elicited after priming with eOD-GT8 60mer. Statistical comparisons were made by Mann-Whitney. *p<0.05, **p<0.01; ns, not significant.

**Fig. S10. LCDR3 logo plots for VRC01-class V_K_1-33 BCRs.** **(A and B)** Logo plots are shown for BCRs analyzed after eOD-GT8 mRNA priming (A) and after core-g28v2 mRNA boosting (B).

**Fig. S11. Comparison of protein plus adjuvant immunization versus immunization with mRNA/LNPs.** Select data repeated from Fig. 2D, 2E, and 2F; Fig. 4C; Fig. 5C and 5D; Fig. 6A; and fig. S8A. **(A)** The frequency of VRC01-class MBCs among total MBCs after priming with eOD-GT8 60mer and boosting with core-g28v2 60mer. **(B)** The frequency of VRC01-class MBCs with human V_K_1-33 light chains among total MBCs after priming with eOD-GT8 60mer and boosting with core-g28v2 60mer. **(C)** Median percent amino acid SHM in the V_H_ gene among all VRC01-class MBCs after priming with eOD-GT8 60mer and boosting with core-g28v2 60mer. **(D)** 90^th^ percentile key VRC01-class heavy chain residues elicited after priming with eOD-GT8 60mer and boosting with core-g28v2 60mer. Statistical comparisons were made by Mann-Whitney. *p<0.05, **p<0.01; ns, not significant.

**Fig. S12. Key VRC01-class heavy chain residues elicited after mRNA/LNP immunization.** **(A and B)** Key VRC01-class heavy chain residues among the GT8 mRNA primed group (A) and the GT8 mRNA primed, placebo boosted group (sorted with GT8) (B). **(C)** Key VRC01-class heavy chain residues among the GT8 mRNA primed, GT8 mRNA boosted group (sorted with GT8). **(D)** Key VRC01-class heavy chain residues among the GT8 mRNA primed, GT8 mRNA boosted group (sorted with core). Colors along the y-axis represent individual mice.

**Fig. S13. SPR/BLI analyses of VRC01-class mAb specificity. (A)** Monovalent K_D_ values measured by SPR for core-g28v2 and core-g28v2-KO to VRC01-class mAbs isolated from SE09 mice after boosting with core-g28v2 60mer mRNA/LNPs. **(B)** VRC01 Fab competition BLI for core-g28v2 versus VRC01-class (VRC01c) and non-VRC01-class (nonVRC01c) mAbs elicited after boosting with core-g28v2 60mer mRNA/LNPs. Data shown across four plots for ease of viewing.

**Fig. S14. SPR analysis of non-VRC01-class mAb affinity and specificity. (A)** Monovalent K_D_ values measured by SPR for eOD-GT8, eOD-GT8-KO, and core-g28v2 to non-VRC01-class mAbs isolated from SE09 mice after priming with eOD-GT8 60mer mRNA/LNPs. **(B)** Monovalent K_D_ values measured by SPR for core-g28v2 and core-g28v2-KO to non-VRC01-class mAbs isolated from SE09 mice after priming with eOD-GT8 60mer mRNA/LNPs and boosting with core-g28v2 60mer mRNA/LNPs. Black lines indicate medians.

**Fig. S15. SHM analysis of non-VRC01-class BCRs induced by mRNA/LNP immunization. (A)** Median percent amino acid SHM in the VH gene among non-VRC01-class MBCs induced after mRNA/LNP immunization. **(B)** Median percent amino acid SHM in the VK/VL genes among non-VRC01-class MBCs induced after mRNA/LNP immunization. Each point represents the median per mouse and the red bars indicate the median of medians.

**Fig. S16. Pseudovirus neutralization activity of polyclonal IgGs isolated from serum after eOD-GT8 60mer mRNA/LNP priming or boosting with core-g28v2 mRNA/LNPs**. Murine leukemia virus (MLV) was used as a negative control. No neutralization: NN; IC_50_ > 50 µg/mL.

Fig. S17. SPR studies of boost candidate interactions with VRC01-class mAbs. (A) SPR affinity data for VRC01-class mAbs elicited by eOD-GT8 60mer protein immunization in the V_H_1-2 mouse model (*22*). (B) SPR affinity data for VRC01-class mAbs elicited by eOD-GT8 60mer protein immunization in humans (*26*) binding to gp120 core variants with or without the T278M mutation. Red lines indicate medians.

Table S1. Immunogen amino acid sequences with signal peptides in blue.

**Data file S1**. Raw, individual-level data.
